# Rare predicted loss-of-function variants of type I IFN immunity genes are associated with life-threatening COVID-19

**DOI:** 10.1186/s13073-023-01173-8

**Published:** 2023-04-05

**Authors:** Daniela Matuozzo, Estelle Talouarn, Astrid Marchal, Peng Zhang, Jeremy Manry, Yoann Seeleuthner, Yu Zhang, Alexandre Bolze, Matthieu Chaldebas, Baptiste Milisavljevic, Adrian Gervais, Paul Bastard, Takaki Asano, Lucy Bizien, Federica Barzaghi, Hassan Abolhassani, Ahmad Abou Tayoun, Alessandro Aiuti, Ilad Alavi Darazam, Luis M. Allende, Rebeca Alonso-Arias, Andrés Augusto Arias, Gokhan Aytekin, Peter Bergman, Simone Bondesan, Yenan T. Bryceson, Ingrid G. Bustos, Oscar Cabrera-Marante, Sheila Carcel, Paola Carrera, Giorgio Casari, Khalil Chaïbi, Roger Colobran, Antonio Condino-Neto, Laura E. Covill, Ottavia M. Delmonte, Loubna El Zein, Carlos Flores, Peter K. Gregersen, Marta Gut, Filomeen Haerynck, Rabih Halwani, Selda Hancerli, Lennart Hammarström, Nevin Hatipoğlu, Adem Karbuz, Sevgi Keles, Christèle Kyheng, Rafael Leon-Lopez, Jose Luis Franco, Davood Mansouri, Javier Martinez-Picado, Ozge Metin Akcan, Isabelle Migeotte, Pierre-Emmanuel Morange, Guillaume Morelle, Andrea Martin-Nalda, Giuseppe Novelli, Antonio Novelli, Tayfun Ozcelik, Figen Palabiyik, Qiang Pan-Hammarström, Rebeca Pérez de Diego, Laura Planas-Serra, Daniel E. Pleguezuelo, Carolina Prando, Aurora Pujol, Luis Felipe Reyes, Jacques G. Rivière, Carlos Rodriguez-Gallego, Julian Rojas, Patrizia Rovere-Querini, Agatha Schlüter, Mohammad Shahrooei, Ali Sobh, Pere Soler-Palacin, Yacine Tandjaoui-Lambiotte, Imran Tipu, Cristina Tresoldi, Jesus Troya, Diederik van de Beek, Mayana Zatz, Pawel Zawadzki, Saleh Zaid Al-Muhsen, Mohammed Faraj Alosaimi, Fahad M. Alsohime, Hagit Baris-Feldman, Manish J. Butte, Stefan N. Constantinescu, Megan A. Cooper, Clifton L. Dalgard, Jacques Fellay, James R. Heath, Yu-Lung Lau, Richard P. Lifton, Tom Maniatis, Trine H. Mogensen, Horst von Bernuth, Alban Lermine, Michel Vidaud, Anne Boland, Jean-François Deleuze, Robert Nussbaum, Amanda Kahn-Kirby, France Mentre, Sarah Tubiana, Guy Gorochov, Florence Tubach, Pierre Hausfater, COVID Human Genetic Effort, Laurent Abel, Saleh Al-Muhsen, Fahd Al-Mulla, Mark S. Anderson, Evangelos Andreakos, Andrés A. Arias, Hagit Baris Feldman, Alexandre Belot, Catherine M. Biggs, Dusan Bogunovic, Anastasiia Bondarenko, Ahmed A. Bousfiha, Petter Brodin, Yenan Bryceson, Carlos D. Bustamante, Samya Chakravorty, John Christodoulou, Murkesh Desai, Beth A. Drolet, Jamila El Baghdadi, Sara Espinosa-Padilla, José Luis Franco, Antoine Froidure, David Hagin, Sarah E. Henrickson, Elena W. Y. Hsieh, Eystein Husebye, Kohsuke Imai, Yuval Itan, Erich D. Jarvis, Timokratis Karamitros, Kai Kisand, Cheng-Lung Ku, Yun Ling, Carrie L. Lucas, László Maródi, Isabelle Meyts, Joshua D. Milner, Kristina Mironska, Tomohiro Morio, Lisa F. P. Ng, Luigi D. Notarangelo, Cliona O’Farrelly, Satoshi Okada, Anna M. Planas, Lluis Quintana-Murci, Laurent Renia, Igor Resnick, Carlos Rodríguez-Gallego, Vanessa Sancho-Shimizu, Anna Sediva, Mikko R. J. Seppänen, Anna Shcherbina, Ondrej Slaby, Andrew L. Snow, Pere Soler-Palacín, András N. Spaan, Ivan Tancevski, Stuart G. Tangye, Sathishkumar Ramaswamy, Stuart E. Turvey, Furkan Uddin, Mohammed J. Uddin, Diederik van de Beek, Donald C. Vinh, Mayana Zatz, Helen C. Su, Jean-Laurent Casanova, COVIDeF Study Group, Serge Bureau, Yannick Vacher, Anne Gysembergh-Houal, Lauren Demerville, Abla Chachoua, Sebastien Abad, Radhiya Abassi, Abdelrafie Abdellaoui, Abdelkrim Abdelmalek, Hendy Abdoul, Helene Abergel, Fariza Abeud, Sophie Abgrall, Noemie Abisror, Marylise Adechian, Nordine Aderdour, Hakeem Farid Admane, Frederic Adnet, Sara Afritt, Helene Agostini, Claire Aguilar, Sophie Agut, Tommaso Francesco Aiello, Marc Ait Kaci, Hafid Ait Oufella, Gokula Ajeenthiravasan, Virginie Alauzy, Fanny Alby-Laurent, Lucie Allard, Marie-Alexandra Alyanakian, Blanca Amador Borrero, Sabrina Amam, Lucile Amrouche, Marc Andronikof, Dany Anglicheau, Nadia Anguel, Djillali Annane, Mohammed Aounzou, Caroline Aparicio, Gladys Aratus, Jean-Benoit Arlet, Jeremy Arzoine, Elisabeth Aslangul, Mona Assefi, Adeline Aubry, Laetitia Audiffred, Etienne Audureau, Christelle Nathalie Auger, Jean-Charles Auregan, Celine Awotar, Sonia Ayllon Milla, Delphine Azan, Laurene Azemar, Billal Azzouguen, Marwa Bachir Elrufaai, Aïda Badsi, Prissile Bakouboula, Coline Balcerowiak, Fanta Balde, Elodie Baldivia, Eliane-Flore Bangamingo, Amandine Baptiste, Fanny Baran-Marszak, Caroline Barau, Nathalie Barget, Flore Baronnet, Romain Barthelemy, Jean-Luc Baudel, Camille Baudry, Elodie Baudry, Laurent Beaugerie, Adel Belamri, Nicolas Belaube, Rhida Belilita, Pierre Bellassen, Rawan Belmokhtar, Isabel Beltran, Ruben Benainous, Mourad Benallaoua, Robert Benamouzig, Amélie Benbara, Jaouad Benhida, Anis Benkhelouf, Jihene Benlagha, Chahinez Benmostafa, Skander Benothmane, Miassa Bentifraouine, Laurence Berard, Quentin Bernier, Enora Berti, Astrid Bertier, Laure Berton, Simon Bessis, Alexandra Beurton, Celine Bianco, Clara Bianquis, Frank Bidar, Philippe Blanche, Clarisse Blayau, Alexandre Bleibtreu, Emmanuelle Blin, Coralie Bloch-Queyrat, Marie-Christophe Boissier, Diane Bollens, Marion Bolzoni, Rudy pierre Bompard, Nicolas Bonnet, Justine Bonnouvrier, Shirmonecrystal Botha, Wissam Boucenna, Fatiha Bouchama, Olivier Bouchaud, Hanane Bouchghoul, Taoueslylia Boudjebla, Noel Boudjema, Catherine Bouffard, Adrien Bougle, Meriem Bouguerra, Leila Bouras, Agnes Bourcier, Anne Bourgarit Durand, Anne Bourrier, Fabrice Bouscarat, Diane Bouvry, Nesrine Bouziri, Ons Bouzrara, Sarah Bribier, Delphine Brugier, Melanie Brunel, Eida Bui, Anne Buisson, Iryna Bukreyeva, Côme Bureau, Jacques Cadranel, Johann Cailhol, Ruxandra Calin, Clara Campos Vega, Pauline Canavaggio, Marta Cancella, Delphine Cantin, Albert Cao, Lionel Carbillon, Nicolas Carlier, Clementine Cassard, Guylaine Castor, Marion Cauchy, Olivier Cha, Benjamin Chaigne, Salima Challal, Karine Champion, Patrick Chariot, Julie Chas, Simon Chauveau, Anthony Chauvin, Clement Chauvin, Nathalie Chavarot, Kamélia Chebbout, Mustapha Cherai, Ilaria Cherubini, Amelie Chevalier, Thibault Chiarabini, Thierry Chinet, Richard Chocron, Pascaline Choinier, Juliette Chommeloux, Christophe Choquet, Laure Choupeaux, Benjamin Chousterman, Dragosmarius Ciocan, Ada Clarke, Gaëlle Clavere, Florian Clavier, Karine Clement, Sebastien Clerc, Yves Cohen, Fleur Cohen, Adrien Cohen, Audrey Coilly, Hester Colboc, Pauline Colin, Magalie Collet, Chloé Comarmond, Emeline Combacon, Alain Combes, Celine Comparon, Jean-Michel Constantin, Hugues Cordel, Anne-Gael Cordier, Adrien Costantini, Nathalie Costedoat Chalumeau, Camille Couffignal, Doriane Coupeau, Alain Creange, Yannie Cuvillier Lamarre, Charlène Da Silveira, Sandrine Dautheville Guibal El Kayani, Nathalie De Castro, Yann De Rycke, Lucie Del Pozo, Quentin Delannoy, Mathieu Delay, Robin Deleris, Juliette Delforge, Laëtitia Delphine, Noemie Demare, Sophie Demeret, Alexandre Demoule, Aurore Deniau, François Depret, Sophie Derolez, Ouda Derradji, Nawal Derridj, Vincent Descamps, Lydia Deschamps, Celine Desconclois, Cyrielle Desnos, Karine Desongins, Robin Dhote, Benjamin Diallo, Morgane Didier, Myriam Diemer, Stephane Diez, Juliette Djadi-Prat, Fatima-Zohra Djamouri Monnory, Siham Djebara, Naoual Djebra, Minette Djietcheu, Hadjer Djillali, Nouara Djouadi, Severine Donneger, Catarina Dos Santos, Nathalie Dournon, Martin Dres, Laura Droctove, Marie Drogrey, Margot Dropy, Elodie Drouet, Valérie Dubosq, Evelyne Dubreucq, Estelle Dubus, Boris Duchemann, Thibault Duchenoy, Emmanuel Dudoignon, Romain Dufau, Florence Dumas, Clara Duran, Emmanuelle Duron, Antoine Durrbach, Claudine Duvivier, Nathan Ebstein, Jihane El Khalifa, Alexandre Elabbadi, Caroline Elie, Gabriel Ernotte, Anne Esling, Martin Etienne, Xavier Eyer, Muriel sarah Fartoukh, Takoua Fayali, Marion Fermaut, Arianna Fiorentino, Souha Fliss, Marie-Céline Fournier, Benjamin Fournier, Hélène Francois, Olivia Freynet, Yvann Frigout, Isaure Fromont, Axelle Fuentes, Thomas Furet, Joris Galand, Marc Garnier, Agnes Gaubert, Stéphane Gaudry, Samuel Gaugain, Damien Gauthier, Maxime Gautier, Sophie Georgin-Lavialle, Daniela Geromin, Mohamed Ghalayini, Bijan Ghaleh, Myriam Ghezal, Aude Gibelin, Linda Gimeno, Benoit Girard, Bénédicte Giroux Leprieur, Doryan Gomes, Elisabete Gomes-Pires, Anne Gouge, Amel Gouja, Helene Goulet, Sylvain Goupil, Jeanne Goupil De Bouille, Julien Gras, Segolene Greffe, Lamiae Grimaldi, Paul Guedeney, Bertrand Guidet, Matthias Guillo, Mariechristelle Gulczynski, Tassadit Hadjam, Didier Haguenauer, Soumeya Hammal, Nadjib Hammoudi, Olivier Hanon, Anarole Harrois, Coraline Hautem, Guillaume Hekimian, Nicholas Heming, Olivier Hermine, Sylvie Ho, Marie Houllier, Benjamin Huot, Tessa Huscenot, Wafa Ibn Saied, Ghilas Ikherbane, Meriem Imarazene, Patrick Ingiliz, Lina Iratni, Stephane Jaureguiberry, Jean-Francois Jean-Marc, Deleena Jeyarajasingham, Pauline Jouany, Veronique Jouis, Clement Jourdaine, Ouifiya Kafif, Rim Kallala, Sandrine Katsahian, Lilit Kelesyan, Vixra Keo, Flora Ketz, Warda Khamis, Enfel Khelili, Mehdi Khellaf, Christy Gaëlla Kotokpo Youkou, Ilias Kounis, Gaelle Kpalma, Jessica Krause, Vincent Labbe, Karine Lacombe, Jean-Marc Lacorte, Anne Gaelle Lafont, Emmanuel Lafont, Lynda Lagha, Lionel Lamhaut, Aymeric Lancelot, Cecilia Landman, Fanny Lanternier, Cecile Larcheveque, Caroline Lascoux Combe, Ludovic Lassel, Benjamin Laverdant, Christophe Lavergne, Jean-Rémi Lavillegrand, Pompilia Lazureanu, Loïc Le Guennec, Lamia Leberre, Claire Leblanc, Marion Leboyer, Francois Lecomte, Marine Lecorre, Romain Leenhardt, Marylou Lefebvre, Bénédicte Lefebvre, Paul Legendre, Anne Leger, Laurence Legros, Justyna Legrosse, Sébastien Lehuunghia, Julien Lemarec, Jeremie Leporrier-Ext, Manon Lesein, Hubert Lesur, Vincent Levy, Albert Levy, Edwige Lopes, Amanda Lopes, Vanessa Lopez, Julien Lopinto, Olivier Lortholary, Badr Louadah, Bénédicte Loze, Marie-Laure Lucas, Axelle Lucasamichi, Liem Binh Luong, Arouna Magazimama-Ext, David Maingret, Lakhdar Mameri, Philippe Manivet, Cylia Mansouri, Estelle Marcault, Jonathan Marey, Nathalie Marin, Clémence Marois, Olivier Martin, Lou Martineau, Cannelle Martinez-Lopez, Pierre Martyniuck, Pauline Mary De Farcy, Nessrine Marzouk, Rafik Masmoudi, Alexandre Mebazaa, Frédéric Mechai, Fabio Mecozzi, Chamseddine Mediouni, Bruno Megarbane, Mohamed Meghadecha, Élodie Mejean, Arsene Mekinian, Nour Mekki Abdelhadi, Rania Mekni, Thinhinan Sabrina Meliti, Breno Melo Lima, Paris Meng, Soraya Merbah, Fadhila Messani, Yasmine Messaoudi, Baboo-Irwinsingh Mewasing, Lydia Meziane, Carole Michelot-Burger, Françoise Mignot, Fadi Hillary Minka, Makoto Miyara, Pierre Moine, Jean-Michel Molina, Anaïs Montegnies-Boulet, Alexandra Monti, Claire Montlahuc, Anne-Lise Montout, Alexandre Moores, Caroline Morbieu, Helene Mortelette, Stéphane Mouly, Rosita Muzaffar, Cherifa Iness Nacerddine, Marine Nadal, Hajer Nadif, Kladoum Nassarmadji, Pierre Natella, Sandrine Ndingamondze, Stefan Neraal, Caroline Nguyen, Bao N’Guyen, Isabelle Nion Larmurier, Luc Nlomenyengue, Nicolas Noel, Hilario Nunes, Edris Omar, Zineb Ouazene, Elise Ouedraogo, Wassila Ouelaa, Anissa Oukhedouma, Yasmina Ould Amara, Herve Oya, Johanna Oziel, Thomas Padilla, Elena Paillaud, Solenne Paiva, Beatrice Parfait, Perrine Parize, Christophe Parizot, Antoine Parrot, Arthur Pavot, Laetitia Peaudecerf, Frédéric Pene, Marion Pepin, Julie Pernet, Claire Pernin, Mylène Petit, Olivier Peyrony, Marie-Pierre Pietri, Olivia Pietri, Marc Pineton De Chambrun, Michelle Pinson, Claire Pintado, Valentine Piquard, Christine Pires, Benjamin Planquette, Sandrine Poirier, Anne-Laure Pomel, Stéphanie Pons, Diane Ponscarme, Annegaelle Pourcelot, Valérie Pourcher, Anne Pouvaret, Florian Prever, Miresta Previlon, Margot Prevost, Marie-Julie Provoost, Cyril Quemeneur, Cédric Rafat, Agathe Rami, Brigitte Ranque, Maurice Raphael, Jean Herle Raphalen, Anna Rastoin, Mathieu Raux, Amani Rebai, Michael Reby, Alexis Regent, Asma Regrag, Matthieu Resche-Rigon, Quentin Ressaire, Christian Richard, Mariecaroline Richard, Maxence Robert, Benjamin Rohaut, Camille Rolland-Debord, Jacques Ropers, Anne-Marie Roque-Afonso, Charlotte Rosso, Mélanie Rousseaux, Nabila Rousseaux, Swasti Roux, Lorène Roux, Claire Rouzaud, Antoine Rozes, Emma Rubenstein, Jean-Marc Sabate, Sheila Sabet, Sophie-Caroline Sacleux, Nathalie Saidenberg Kermanach, Faouzi Saliba, Dominique Salmon, Laurent Savale, Guillaume Savary, Rebecca Sberro, Anne Scemla, Frederic Schlemmer, Mathieu Schwartz, Saïd Sedfi, Samia Sefir-Kribel, Philippe Seksik, Pierre Sellier, Agathe Selves, Nicole Sembach, Luca Semerano, Marie-Victoire Senat, Damien Sene, Alexandra Serris, Lucile Sese, Naima Sghiouar, Johanna Sigaux, Martin Siguier, Johanne Silvain, Noémie Simon, Tabassome Simon, Lina Innes Skandri, Miassa Slimani, Aurélie Snauwaert, Harry Sokol, Heithem Soliman, Nisrine Soltani, Benjamin Soyer, Gabriel Steg, Lydia Suarez, Tali-Anne Szwebel, Kossi Taffame, Claire Tantet, Mariagrazia Tateo, Igor Theodose, Pierre clement Thiebaud, Caroline Thomas, Kelly Tiercelet, Julie Tisserand, Carole Tomczak, Krystel Torelino, Fatima Touam-Ext, Lilia Toumi, Gustave Toury, Mireille Toy-Miou, Olivia Tran Dinh Thanh Lien, Alexy Trandinh, Jean-Marc Treluyer, Baptiste Trinque, Jennifer Truchot, Simone Tunesi, Matthieu Turpin, Agathe Turpin, Tomas Urbina, Rafael Usubillaga Narvaez, Yurdagul Uzunhan, Prabakar Vaittinadaayar, Arnaud Valent, Maelle Valentian, Nadia Valin, Hélène Vallet, Marina Vaz, Miguel-Alejandro Vazquezibarra, Benoit Vedie, Laetitia Velly, Celine Verstuyft, Cedric Viallette, Eric Vicaut, Dorothee Vignes, Damien Vimpere, Myriam Virlouvet, Guillaume Voiriot, Lena Voisot, Emmanuel Weiss, Nicolas Weiss, Anaïs Winchenne, Youri Yordanov, Lara Zafrani, Mohamad Zaidan, Wissem Zaidi, Cathia Zak, Aida Zarhrate-Ghoul, Ouassila Zatout, Suzanne Zeino, Michel Zeitouni, Naïma Zemirli, Lorene Zerah, Ounsa Zia, Marianne Ziol, Oceane Zolario, Julien Zuber, French COVID Cohort Study Group, Laurent Abel, Claire Andrejak, François Angoulvant, Delphine Bachelet, Marie Bartoli, Romain Basmaci, Sylvie Behilill, Marine Beluze, Dehbia Benkerrou, Krishna Bhavsar, Lila Bouadma, Sabelline Bouchez, Maude Bouscambert, Minerva Cervantes-Gonzalez, Anissa Chair, Catherine Chirouze, Alexandra Coelho, Camille Couffignal, Sandrine Couffin-Cadiergues, Eric d’Ortenzio, Marie-Pierre Debray, Lauren Deconinck, Dominique Deplanque, Diane Descamps, Mathilde Desvallée, Alpha Diallo, Alphonsine Diouf, Céline Dorival, François Dubos, Xavier Duval, Brigitte Elharrar, Philippine Eloy, Vincent Enouf, Hélène Esperou, Marina Esposito-Farese, Manuel Etienne, Eglantine Ferrand Devouge, Nathalie Gault, Alexandre Gaymard, Jade Ghosn, Tristan Gigante, Morgane Gilg, Jérémie Guedj, Alexandre Hoctin, Isabelle Hoffmann, Ikram Houas, Jean-Sébastien Hulot, Salma Jaafoura, Ouifiya Kafif, Florentia Kaguelidou, Sabrina Kali, Antoine Khalil, Coralie Khan, Cédric Laouénan, Samira Laribi, Minh Le, Quentin Le Hingrat, Soizic Le Mestre, Hervé Le Nagard, François-Xavier Lescure, Sophie Letrou, Yves Levy, Bruno Lina, Guillaume Lingas, Jean Christophe Lucet, Denis Malvy, Marina Mambert, France Mentré, Amina Meziane, Hugo Mouquet, Jimmy Mullaert, Nadège Neant, Duc Nguyen, Marion Noret, Saad Nseir, Aurélie Papadopoulos, Christelle Paul, Nathan Peiffer-Smadja, Thomas Perpoint, Ventzislava Petrov-Sanchez, Gilles Peytavin, Huong Pham, Olivier Picone, Valentine Piquard, Oriane Puéchal, Christian Rabaud, Manuel Rosa-Calatrava, Bénédicte Rossignol, Patrick Rossignol, Carine Roy, Marion Schneider, Richa Su, Coralie Tardivon, Marie-Capucine Tellier, François Téoulé, Olivier Terrier, Jean-François Timsit, Christelle Tual, Sylvie Van Der Werf, Noémie Vanel, Aurélie Veislinger, Benoit Visseaux, Aurélie Wiedemann, Yazdan Yazdanpanah, CoV-Contact Cohort, Loubna Alavoine, Sylvie Behillil, Charles Burdet, Charlotte Charpentier, Aline Dechanet, Diane Descamps, Xavier Duval, Jean-Luc Ecobichon, Vincent Enouf, Wahiba Frezouls, Nadhira Houhou, Ouifiya Kafif, Jonathan Lehacaut, Sophie Letrou, Bruno Lina, Jean-Christophe Lucet, Pauline Manchon, Mariama Nouroudine, Valentine Piquard, Caroline Quintin, Michael Thy, Sylvie van der Werf, Valérie Vignali, Benoit Visseaux, Yazdan Yazdanpanah, Abir Chahine, Nawal Waucquier, Maria-Claire Migaud, Dominique Deplanque, Félix Djossou, Mayka Mergeay-Fabre, Aude Lucarelli, Magalie Demar, Léa Bruneau, Patrick Gérardin, Adrien Maillot, Christine Payet, Bruno Laviolle, Fabrice Laine, Christophe Paris, Mireille Desille-Dugast, Julie Fouchard, Denis Malvy, Duc Nguyen, Thierry Pistone, Pauline Perreau, Valérie Gissot, Carole L. E. Goas, Samatha Montagne, Lucie Richard, Catherine Chirouze, Kévin Bouiller, Maxime Desmarets, Alexandre Meunier, Marilou Bourgeon, Benjamin Lefévre, Hélène Jeulin, Karine Legrand, Sandra Lomazzi, Bernard Tardy, Amandine Gagneux-Brunon, Frédérique Bertholon, Elisabeth Botelho-Nevers, Kouakam Christelle, Leturque Nicolas, Layidé Roufai, Karine Amat, Sandrine Couffin-Cadiergues, Hélène Espérou, Samia Hendou, COVID-STORM Clinicians, Giuseppe Foti, Giacomo Bellani, Giuseppe Citerio, Ernesto Contro, Alberto Pesci, Maria Grazia Valsecchi, Marina Cazzaniga, COVID Clinicians, Jorge Abad, Giulia Accordino, Cristian Achille, Sergio Aguilera-Albesa, Aina Aguiló-Cucurull, Esra Akyüz Özkan, Jonathan Antonio Roblero Albisures, Juan C. Aldave, Miquel Alfonso Ramos, Taj Ali Khan, Anna Aliberti, Seyed Alireza Nadji, Gulsum Alkan, Suzan A. AlKhater, Jerome Allardet-Servent, Mohammed S. Alshahrani, Laia Alsina, Marie-Alexandra Alyanakian, Blanca Amador Borrero, Zahir Amoura, Arnau Antolí, Romain Arrestier, Mélodie Aubart, Teresa Auguet, Iryna Avramenko, Gökhan Aytekin, Axelle Azot, Seiamak Bahram, Fanny Bajolle, Fausto Baldanti, Aurélie Baldolli, Maite Ballester, Hagit Baris Feldman, Benoit Barrou, Sabrina Basso, Gulsum Iclal Bayhan, Alexandre Belot, Liliana Bezrodnik, Agurtzane Bilbao, Geraldine Blanchard-Rohner, Ignacio Blanco, Adeline Blandinières, Daniel Blázquez-Gamero, Alexandre Bleibtreu, Marketa Bloomfield, Mireia Bolivar-Prados, Anastasiia Bondarenko, Alessandro Borghesi, Raphael Borie, Elisabeth Botdhlo-Nevers, Ahmed A. Bousfiha, Aurore Bousquet, David Boutolleau, Claire Bouvattier, Oksana Boyarchuk, Juliette Bravais, M. Luisa Briones, Marie-Eve Brunner, Raffaele Bruno, Maria Rita P. Bueno, Huda Bukhari, Jacinta Bustamante, Juan José Cáceres Agra, Ruggero Capra, Raphael Carapito, Maria Carrabba, Carlos Casasnovas, Marion Caseris, Irene Cassaniti, Martin Castelle, Francesco Castelli, Martín Castillo de Vera, Mateus V. Castro, Emilie Catherinot, Jale Bengi Celik, Alessandro Ceschi, Martin Chalumeau, Bruno Charbit, Matthew P. Cheng, Père Clavé, Bonaventura Clotet, Anna Codina, Yves Cohen, Cloé Comarmond, Alain Combes, Patrizia Comoli, Angelo G. Corsico, Betul Sozeri, Taner Coşkuner, Aleksandar Cvetkovski, Cyril Cyrus, David Dalmau, François Danion, David Ross Darley, Vincent Das, Nicolas Dauby, Stéphane Dauger, Paul De Munter, Loic de Pontual, Amin Dehban, Geoffroy Delplancq, Alexandre Demoule, Isabelle Desguerre, Antonio Di Sabatino, Jean-Luc Diehl, Stephanie Dobbelaere, Elena Domínguez-Garrido, Clément Dubost, Olov Ekwall, Şefika Elmas Bozdemir, Marwa H. Elnagdy, Melike Emiroglu, Akifumi Endo, Emine Hafize Erdeniz, Selma Erol Aytekin, Maria Pilar Etxart Lasa, Romain Euvrard, Giovanna Fabio, Laurence Faivre, Antonin Falck, Muriel Fartoukh, Morgane Faure, Miguel Fernandez Arquero, Ricard Ferrer, Jose Ferreres, Bruno Francois, Victoria Fumadó, Kitty S. C. Fung, Francesca Fusco, Alenka Gagro, Blanca Garcia Solis, Pierre Garçon, Pascale Gaussem, Zeynep Gayretli, Juana Gil-Herrera, Laurent Gilardin, Audrey Giraud Gatineau, Mònica Girona-Alarcón, Karen Alejandra Cifuentes Godínez, Jean-Christophe Goffard, Nacho Gonzales, Luis I. Gonzalez-Granado, Rafaela González-Montelongo, Antoine Guerder, Belgin Gülhan, Victor Daniel Gumucio, Leif Gunnar Hanitsch, Jan Gunst, Jérôme Hadjadj, Tetyana Hariyan, Nevin Hatipoglu, Deniz Heppekcan, Elisa Hernandez-Brito, Po-ki Ho, María Soledad Holanda-Peña, Juan P. Horcajada, Sami Hraiech, Linda Humbert, Ivan F. N. Hung, Alejandro D. Iglesias, Antonio Íñigo-Campos, Matthieu Jamme, María Jesús Arranz, Marie-Thérèse Jimeno, Iolanda Jordan, Saliha Kanik-Yüksek, Yalcin Burak Kara, Aydın Karahan, Kadriye Kart Yasar, Ozgur Kasapcopur, Kenichi Kashimada, Yasemin Kendir Demirkol, Yasutoshi Kido, Can Kizil, Ahmet Osman Kılıç, Adam Klocperk, Antonia Koutsoukou, Zbigniew J. Król, Hatem Ksouri, Paul Kuentz, Arthur M. C. Kwan, Yat Wah M. Kwan, Janette S. Y. Kwok, Jean-Christophe Lagier, David S. Y. Lam, Vicky Lampropoulou, Fanny Lanternier, Fleur Le Bourgeois, Yee-Sin Leo, Rafael Leon Lopez, Daniel Leung, Michael Levin, Michael Levy, Romain Lévy, Zhi Li, Daniele Lilleri, Edson Jose Adrian Bolanos Lima, Agnes Linglart, Eduardo López-Collazo, José M. Lorenzo-Salazar, Céline Louapre, Catherine Lubetzki, Kwok-Cheung Lung, Charles-Edouard Luyt, David C. Lye, Cinthia Magnone, Enrico Marchioni, Carola Marioli, Majid Marjani, Laura Marques, Jesus Marquez Pereira, Andrea Martín-Nalda, David Martínez Pueyo, Iciar Marzana, Carmen Mata-Martínez, Alexis Mathian, Larissa R. B. Matos, Gail V. Matthews, Julien Mayaux, Raquel McLaughlin-Garcia, Philippe Meersseman, Jean-Louis Mège, Armand Mekontso-Dessap, Isabelle Melki, Federica Meloni, Jean-François Meritet, Paolo Merlani, Özge Metin Akcan, Isabelle Meyts, Mehdi Mezidi, Maude Millereux, Matthieu Million, Tristan Mirault, Clotilde Mircher, Mehdi Mirsaeidi, Yoko Mizoguchi, Bhavi P. Modi, Francesco Mojoli, Elsa Moncomble, Abián Montesdeoca Melián, Antonio Morales Martinez, Francisco Morandeira, Cléemence Mordacq, Stéphane J. Mouly, Adrián Muñoz-Barrera, Cyril Nafati, Shintaro Nagashima, Yu Nakagama, Bénédicte Neven, João Farela Neves, Lisa F. P. Ng, Yuk-Yung Ng, hubert Nielly, Yeray Novoa Medina, Esmeralda Nuñez Cuadros, J. Gonzalo Ocejo-Vinyals, Keisuke Okamoto, Mehdi Oualha, Amani Ouedrani, Tayfun Özçelik, Aslinur Ozkaya-Parlakay, Michele Pagani, Maria Papadaki, Christophe Parizot, Philippe Parola, Tiffany Pascreau, Stéphane Paul, Estela Paz-Artal, Sigifredo Pedraza, Nancy Carolina González Pellecer, Silvia Pellegrini, Xosé Luis Pérez-Fernández, Aurélien Philippe, Quentin Philippot, Adrien Picod, Marc Pineton de Chambrun, Antonio Piralla, Dominique Ploin, Julien Poissy, Géraldine Poncelet, Garyphallia Poulakou, Marie S. Pouletty, Persia Pourshahnazari, Jia Li Qiu-Chen, Paul Quentric, Thomas Rambaud, Didier Raoult, Violette Raoult, Anne-Sophie Rebillat, Claire Redin, Léa Resmini, Pilar Ricart, Jean-Christophe Richard, Raúl Rigo-Bonnin, Nadia rivet, Gemma Rocamora-Blanch, Mathieu P. Rodero, Carlos Rodrigo, Luis Antonio Rodriguez, Agustí Rodriguez-Palmero, Carolina Soledad Romero, Anya Rothenbuhler, Damien Roux, Nikoletta Rovina, Flore Rozenberg, Yvon Ruch, Montse Ruiz, Maria Yolanda Ruiz del Prado, Juan Carlos Ruiz-Rodriguez, Joan Sabater-Riera, Kai Saks, Maria Salagianni, Oliver Sanchez, Adrián Sánchez-Montalvá, Silvia Sánchez-Ramón, Laire Schidlowski, Agatha Schluter, Julien Schmidt, Matthieu Schmidt, Catharina Schuetz, Cyril E. Schweitzer, Francesco Scolari, Anna Sediva, Luis Seijo, Analia Gisela Seminario, Damien Sene, Piseth Seng, Sevtap Senoglu, Mikko Seppänen, Alex Serra Llovich, Anna Shcherbina, Virginie Siguret, Eleni Siouti, David M. Smadja, Nikaia Smith, Xavier Solanich, Jordi Solé-Violán, Catherine Soler, Pere Soler-Palacín, Betül Sözeri, Giulia Maria Stella, Yuriy Stepanovskiy, Annabelle Stoclin, Fabio Taccone, Jean-Luc Taupin, Simon J. Tavernier, Loreto Vidaur Tello, Benjamin Terrier, Guillaume Thiery, Christian Thorball, Karolina Thorn, Caroline Thumerelle, Martin Tolstrup, Gabriele Tomasoni, Julie Toubiana, Josep Trenado Alvarez, Vasiliki Triantafyllia, Sophie Trouillet-Assant, Jesús Troya, Owen T. Y. Tsang, Liina Tserel, Eugene Y. K. Tso, Alessandra Tucci, Şadiye Kübra Tüter Öz, Matilde Valeria Ursini, Takanori Utsumi, Yurdagul Uzunhan, Pierre Vabres, Juan Valencia-Ramos, Ana Maria Van Den Rym, Isabelle Vandernoot, Valentina Velez-Santamaria, Silvia Patricia Zuniga Veliz, Mateus C. Vidigal, Sébastien Viel, Cédric Villain, Marie E. Vilaire-Meunier, Judit Villar-García, Audrey Vincent, Guillaume Voiriot, Alla Volokha, Fanny Vuotto, Els Wauters, Joost Wauters, Alan K. L. Wu, Tak-Chiu Wu, Aysun Yahşi, Osman Yesilbas, Mehmet Yildiz, Barnaby E. Young, Ufuk Yükselmiş, Mayana Zatz, Marco Zecca, Valentina Zuccaro, Jens Van Praet, Bart N. Lambrecht, Eva Van Braeckel, Cédric Bosteels, Levi Hoste, Eric Hoste, Fré Bauters, Jozefien De Clercq, Catherine Heijmans, Hans Slabbynck, Leslie Naesens, Benoit Florkin, Cécile Boulanger, Dimitri Vanderlinden, Orchestra Working Group, Laurent Abel, Matilda Berkell, Valerio Carelli, Alessio Fiorentino, Surbi Malhotra, Alessandro Mattiaccio, Tommaso Pippucci, Marco Seri, Evelina Tacconelli, Amsterdam UMC Covid-19 Biobank, Michiel van Agtmael, Anne Geke Algera, Brent Appelman, Frank van Baarle, Diane Bax, Martijn Beudel, Harm Jan Bogaard, Marije Bomers, Peter Bonta, Lieuwe Bos, Michela Botta, Justin de Brabander, Godelieve de Bree, Sanne de Bruin, David T. P. Buis, Marianna Bugiani, Esther Bulle, Osoul Chouchane Alex Cloherty, Mirjam Dijkstra, Dave A. Dongelmans, Romein W. G. Dujardin, Paul Elbers, Lucas Fleuren, Suzanne Geerlings Theo Geijtenbeek, Armand Girbes, Bram Goorhuis, Martin P. Grobusch, Florianne Hafkamp, Laura Hagens, Jorg Hamann, Vanessa Harris, Robert Hemke, Sabine M. Hermans Leo Heunks, Markus Hollmann, Janneke Horn, Joppe W. Hovius, Menno D. de Jong, Rutger Koning, Endry H. T. Lim, Niels van Mourik, Jeaninne Nellen, Esther J. Nossent, Frederique Paulus, Edgar Peters, Dan A. I. Pina-Fuentes, Tom van der Poll, Bennedikt Preckel, Jan M. Prins, Jorinde Raasveld, Tom Reijnders, Maurits C. F. J. de Rotte, Michiel Schinkel, Marcus J. Schultz, Femke A. P. Schrauwen, Alex Schuurmans, Jaap Schuurmans, Kim Sigaloff, Marleen A. Slim, Patrick Smeele, Marry Smit, Cornelis S. Stijnis, Willemke Stilma, Charlotte Teunissen, Patrick Thoral, Anissa M. Tsonas, Pieter R. Tuinman, Marc van der Valk, Denise Veelo, Carolien Volleman, Heder de Vries, Lonneke A. Vught, Michèle van Vugt, Dorien Wouters, A. H. Zwinderman, Matthijs C. Brouwer, W. Joost Wiersinga, Alexander P. J. Vlaar, Diederik van de Beek, NIAID-USUHS COVID Study Group, Miranda F. Tompkins, Camille Alba, Andrew L. Snow, Daniel N. Hupalo, John Rosenberger, Gauthaman Sukumar, Matthew D. Wilkerson, Xijun Zhang, Justin Lack, Andrew J. Oler, Kerry Dobbs, Jeffrey J. Danielson, Andrea Biondi, Laura Rachele Bettini, Mariella D’Angio’, Ilaria Beretta, Luisa Imberti, Alessandra Sottini, Virginia Quaresima, Eugenia Quiros-Roldan, Camillo Rossi, Isabelle Meyts, Shen-Ying Zhang, Anne Puel, Luigi D. Notarangelo, Stephanie Boisson-Dupuis, Helen C. Su, Bertrand Boisson, Emmanuelle Jouanguy, Jean-Laurent Casanova, Qian Zhang, Laurent Abel, Aurélie Cobat

**Affiliations:** 1 Laboratory of Human Genetics of Infectious Diseases, Necker Branch, INSERM U1163, Paris, France; 2 University Paris Cité, Imagine Institute, Paris, France; 3 St. Giles Laboratory of Human Genetics of Infectious Diseases, Rockefeller Branch, The Rockefeller University, New York, NY USA; 4 Laboratory of Clinical Immunology and Microbiology, Division of Intramural Research, NIAID, Bethesda, MD USA; 5 Helix, San Mateo, CA USA; 6 Pediatric Hematology-Immunology and Rheumatology Unit, Necker Hospital for Sick Children, Assistance Publique-Hôpitaux de Paris (AP-HP), Paris, France; 7 Department of Paediatric Immunohematology, IRCCS San Raffaele Scientific Institute, Milan, Italy; 8 Department of Biosciences and Nutrition, Karolinska Institute, Stockholm, Sweden; 9 Research Center for Immunodeficiencies, Pediatrics Center of Excellence, Children’s Medical Center, Tehran, Iran; 10 Genomics Center of Excellence, Al Jalila Children’s Specialty Hospital, Dubai, United Arab Emirates; 11 Center for Genomic Discovery, Mohammed Bin Rashid University of Medicine and Health Sciences, Dubai, United Arab Emirates; 12 San Raffaele Telethon Institute for Gene Therapy (SR-Tiget), IRCSS San Raffaele Scientific Institute, Milan, Italy; 13 Vita-Salute San Raffaele University, Milan, Italy; 14 Infectious Diseases and Tropical Medicine Research Center, Shahid Beheshti University of Medical Sciences, Tehran, Iran; 15 Department of Infectious Diseases and Tropical Medicine, Loghman Hakim Hospital, Shahid Beheshti University of Medical Sciences, Tehran, Iran; 16 Immunology Department, University Hospital 12 de Octubre, Research Institute imas12 and Complutense University, Madrid, Spain; 17 Immunology Department, Hospital Universitario Central de Asturias; Health Research Institute of Principality of Asturias, Oviedo, Spain; 18 Department of Microbiology and Parasitology, Primary Immunodeficiencies Group, School of Medicine, University of Antioquia UdeA, 050010 Medellin, Colombia; 19 School of Microbiology, University of Antioquia UdeA, 050010 Medellin, Colombia; 20 Deparment of Internal Medicine, Division of Allergy and Immunology, Konya City Hospital, Konya, Turkey; 21 Department of Infectious Diseases, The Immunodeficiency Unit, Karolinska University Hospital, Stockholm, Sweden; 22 Department of Laboratory Medicine, Division of Clinical Immunology, Stockholm, Sweden; 23 Clinical Genomics, IRCSS San Raffaele Scientific Institute, Milan, Italy; 24 Department of Medicine, Centre for Hematology and Regenerative Medicine, Karolinska Institute, Stockholm, Sweden; 25 Universidad de La Sabana, Chía, Colombia; 26 Institute of Biomedical Research of IdiPAZ, University Hospital “La Paz”, Madrid, Spain; 27 Unidad de Gestión Clínica de Cuidados Intensivos, Instituto Maimónides de Investigación Biomédica de Córdoba (IMIBIC), Hospital Universitario Reina Sofía, Universidad de Córdoba (UCO), Córdoba, Spain; 28 Division of Genetics and Cell Biology, Genome-Phenome Relationship, San Raffaele Hospital, Milan, Italy; 29 School of Medicine, Vita-Salute San Raffaele University, Milan, Italy; 30 Intensive Care Unit Department, Avicenne Hospital, Assistance Publique-Hôpitaux de Paris, Bobigny, France; 31 Common and Rare Kidney Diseases, Sorbonne University, INSERM UMR-S 1155, Paris, France; 32 Jeffrey Modell Diagnostic and Research Center for Primary Immunodeficiencies, Barcelona, Catalonia Spain; 33 Translational Immunology Research Group, Vall d’Hebron Research Institute (VHIR), Vall d’Hebron University Hospital (HUVH), Vall d’Hebron Barcelona Hospital Campus, Barcelona, Catalonia Spain; 34 Genetics Department, Immunology Division, Vall d’Hebron University Hospital (HUVH), Vall d’Hebron Barcelona Hospital Campus, Autonomous University of Barcelona (UAB), Barcelona, Catalonia Spain; 35 Department of Immunology, Institute of Biomedical Sciences, University of São Paulo, São Paulo, Brazil; 36 Biology Department, Lebanese University, Beirut, Lebanon; 37 Genomics Division, Institute of Technology and Renewable Energies (ITER), Santa Cruz de Tenerife, Spain; 38 CIBER de Enfermedades Respiratorias, Carlos III Health Institute, Madrid, Spain; 39 Research Unit, University Hospital of Ntra. Sra. de Candelaria, Santa Cruz de Tenerife, Spain; 40 Faculty of Health Sciences, University of Fernando Pessoa Canarias, Las Palmas de Gran Canaria, Spain; 41 Feinstein Institute for Medical Research, Northwell Health USA, Manhasset, NY USA; 42 CNAG-CRG, Centre for Genomic Regulation (CRG), The Barcelona Institute of Science and Technology (BIST), Barcelona, Spain; 43 Department of Internal Diseases and Pediatrics, Primary Immune Deficiency Research Laboratory, Centre for Primary Immunodeficiency Ghent, Jeffrey Modell Diagnosis and Research Centre, Ghent, Belgium; 44 Research Institute for Medical and Health Sciences, University of Sharjah, Sharjah, United Arab Emirates; 45 Department of Pediatrics (Infectious Diseases), Istanbul Faculty of Medicine, Istanbul University, Istanbul, Turkey; 46 Pediatric Infectious Diseases Unit, Bakirkoy Dr Sadi Konuk Training and Research Hospital, University of Health Sciences, Istanbul, Turkey; 47 Department of Pediatric Infectious Disease, Dr. Cemil Tascioglu City Hospital, Istanbul, Turkey; 48 Meram Medical Faculty, Pediatric Infectious Diseases Department, Necmettin Erbakan University, Konya, Turkey; 49 Department of General Paediatrics, Hôpital Bicêtre, Assistance Publique-Hôpitaux de Paris, University of Paris Saclay, Le Kremlin-Bicêtre, France; 50 Primary Immunodeficiencies Group, Department of Microbiology and Parasitology, School of Medicine, University of Antioquia UDEA, Medellin, 050010 Colombia; 51 The Clinical Tuberculosis and Epidemiology Research Center, National Research Institute of Tuberculosis and Lung Diseases, Shahid Beheshti University of Medical Sciences, Tehran, Iran; 52 Department of Clinical Immunology and Infectious Diseases, National Research Institute of Tuberculosis and Lung Diseases, Shahid Beheshti University of Medical Sciences, Tehran, Iran; 53 Pediatric Respiratory Diseases Research Center, National Research Institute of Tuberculosis and Lung Diseases, Shahid Beheshti University of Medical Sciences, Tehran, Iran; 54 IrsiCaixa AIDS Research Institute and Institute for Health Science Research Germans Trias I Pujol (IGTP), Badalona, Spain; 55 Institute for Health Science Research Germans Trias I Pujol (IGTP), Badalona, Spain; 56 Department of Infectious Diseases and Immunity, University of Vic-Central University of Catalonia, Vic, Spain; 57 Catalan Institution of Research and Advanced Studies (ICREA), Barcelona, Spain; 58 Consorcio Centro de Investigación Biomédica en Red de Enfermedades Infecciosas (CIBERINFEC), Instituto de Salud Carlos III, Madrid, Spain; 59 Centre de Génétique Humaine de L’Université Libre de Bruxelles, Hôpital Erasme, Brussels, Belgium; 60 Laboratory of Haematology, La Timone Hospital, Marseille, France; 61 C2VN, INSERM, INRAE, Aix-Marseille University, Marseille, France; 62 Pediatric Infectious Diseases and Immunodeficiencies Unit, Vall d’Hebron Research Institute (VHIR), Vall d’Hebron University Hospital (HUVH), Vall d’Hebron Barcelona Hospital Campus, Autonomous University of Barcelona (UAB), Barcelona, Catalonia Spain; 63 Infection and Immunity in Pediatric Patients Research Group, Vall d’Hebron Research Institute (VHIR), Vall d’Hebron University Hospital (HUVH), Vall d’Hebron Barcelona Hospital Campus, Barcelona, Catalonia Spain; 64 Department of Biomedicine and Prevention, Tor Vergata University of Rome, Rome, Italy; 65 IRCCS Neuromed, Pozzilli, Italy; 66 Laboratory of Medical Genetics, Translational Cytogenomics Research Unit, Bambino Gesù Children Hospital, IRCCS, Rome, Italy; 67 Department of Molecular Biology and Genetics, Bilkent University, Ankara, Turkey; 68 Laboratory of Immunogenetics of Human Diseases, IdiPAZ Institute for Health Research, University Hospital “La Paz”, Madrid, Spain; 69 Neurometabolic Diseases Laboratory, Bellvitge Biomedical Research Institute (IDIBELL), Barcelona, Catalonia Spain; 70 Center for Biomedical Research On Rare Diseases (CIBERER), ISCIII, Madrid, Spain; 71 Faculdades Pequeno Príncipe, Instituto de Pesquisa Pelé Pequeno Príncipe, Curitiba, Brazil; 72 Universidad de La Sabana, Chía, Colombia; 73 Department of Immunology, University Hospital of Gran Canaria Dr. Negrin, Canarian Health System, Las Palmas de Gran Canaria, Spain; 74 Department of Clinical Sciences, University of Fernando Pessoa Canarias, Las Palmas de Gran Canaria, Spain; 75 Division of Immunology, Transplantation and Infectious Diseases, IRCCS San Raffaele Scientific Institute, Milan, Italy; 76 Specialized Immunology Laboratory of Dr Shahrooei, Sina Medical Complex, Ahvaz, Iran; 77 Department of Microbiology and Immunology, Clinical and Diagnostic Immunology, KU Leuven, Leuven, Belgium; 78 Department of Pediatrics, Mansoura University Children’s Hospital, Mansoura University Faculty of Medicine, Mansoura, Egypt; 79 Hypoxia and Lung, INSERM U1272, Avicenne Hospital, Assistance Publique-Hôpitaux de Paris, Bobigny, France; 80 Department of Life Sciences, School of Science, University of Management and Technology, Lahore, Pakistan; 81 Division of Immunology, Transplantation and Infectious Diseases, IRCCS Ospedale San Raffaele, Milan, Italy; 82 Department of Internal Medicine, Infanta Leonor University Hospital, Madrid, Spain; 83 Department of Neurology, Amsterdam UMC, Amsterdam Neuroscience, Amsterdam, Netherlands; 84 Biosciences Institute, University of São Paulo, São Paulo, Brazil; 85 Gordion Bioscience Inc, Cambridge, MA USA; 86 Faculty of Physics, Adam Mickiewicz University, Poznan, Poland; 87 Department of Pediatrics, Immunology Research Laboratory, College of Medicine and King Saud University Medical City, King Saud University, Riyadh, Saudi Arabia; 88 The Genetics Institute, Tel Aviv Sourasky Medical Center, Tel Aviv, Israel; 89 Sackler Faculty of Medicine, Tel Aviv University, Tel Aviv, Israel; 90 Departments of Pediatrics and Microbiology, Immunology, and Molecular Genetics, Division of Immunology, Allergy, and Rheumatology, University of California Los Angeles, Los Angeles, CA USA; 91 Ludwig Institute for Cancer Research, Brussels, Belgium; 92 SIGN Unit, de Duve Institute, Université Catholique de Louvain, Brussels, Belgium; 93 WELBIO (Walloon Excellence in Life Sciences and Biotechnology), Brussels, Belgium; 94 Nuffield Department of Medicine, Ludwig Institute for Cancer Research, Oxford University, Oxford, UK; 95 Department of Pediatrics, Division of Rheumatology/Immunology, Washington University in St. Louis, St. Louis, MO USA; 96 The American Genome Center, Uniformed Services University of the Health Sciences, Bethesda, USA; 97 Department of Anatomy, Physiology & Genetics, Uniformed Services University of the Health Sciences, Bethesda, MD USA; 98 School of Life Sciences, École Polytechnique Fédérale de Lausanne, Lausanne, Switzerland; 99 Swiss Institute of Bioinformatics, Lausanne, Switzerland; 100 Precision Medicine Unit, Lausanne University Hospital and University of Lausanne, Lausanne, Switzerland; 101 Institute for Systems Biology, Seattle, WA USA; 102 Department of Paediatrics and Adolescent Medicine, Queen Mary Hospital, Li Ka Shing Faculty of Medicine, University of Hong Kong, Hong Kong, China; 103 Laboratory of Genetics and Genomics, The Rockefeller University, New York, NY USA; 104 Department of Genetics, Yale University School of Medicine, New Haven, CT USA; 105 Yale Center for Genome Analysis, Yale School of Medicine, New Haven, CT USA; 106 Zukerman Mind Brain Behavior Institute, Columbia University, New York, NY USA; 107 New York Genome Center, New York, NY USA; 108 Department of Biomedicine, Aarhus University, Aarhus, Denmark; 109 Department of Infectious Diseases, Aarhus University Hospital, Aarhus, Denmark; 110 Department of Paediatric Respiratory Medicine, Immunology, and Critical Care Medicine, Charité Universitätsmedizin Berlin, Berlin, Germany; 111 Laboratoire de Biologie Médicale Multisites Seqoia, MG2025, MG2025 Paris, France; 112 Université Paris-Saclay, CEA, Centre National de Recherche en Génomique Humaine (CNRGH), Evry, France; 113 Invitae, San Francisco, CA USA; 114 Unité de Recherche Clinique, Hôpital Bichat, Assistance Publique-Hôpitaux de Paris, Paris, France; 115 Centre d’Investigation Clinique, Hôpital Bichat, Assistance Publique-Hôpitaux de Paris, Paris, France; 116 Sorbonne Université, INSERM Centre d’Immunologie et des Maladies Infectieuses, CIMI-Paris, Département d’immunologie Hôpital Pitié-Salpêtrière, Assistance Publique-Hôpitaux de Paris, Paris, France; 117 Sorbonne Université, INSERM, Institut Pierre Louis d’Epidémiologie et de Santé Publique, AP-HP, Hôpital Pitié Salpêtrière, Département de Santé Publique, Unité de Recherche Clinique PSL-CFX , CIC-1901, Paris, France; 118 Emergency Department, Hôpital Pitié-Salpêtrière, APHP-Sorbonne Université, Paris, France; 119 GRC-14 BIOFAST Sorbonn Université, UMR INSERM 1135, CIMI, Sorbonne Université, Paris, France; 120 Department of Microbiology, Immunology and Transplantation, Laboratory for Inborn Errors of Immunity, KU Leuven, Leuven, Belgium; 121 Laboratory of Host Defenses, NIAID, National Institutes of Health, Bethesda, MA USA; 122 Howard Hughes Medical Institute, New York, NY USA

**Keywords:** Rare variants, COVID-19, Immunity, Type I interferon

## Abstract

**Background:**

We previously reported that impaired type I IFN activity, due to inborn errors of TLR3- and TLR7-dependent type I interferon (IFN) immunity or to autoantibodies against type I IFN, account for 15–20% of cases of life-threatening COVID-19 in unvaccinated patients. Therefore, the determinants of life-threatening COVID-19 remain to be identified in ~ 80% of cases.

**Methods:**

We report here a genome-wide rare variant burden association analysis in 3269 unvaccinated patients with life-threatening COVID-19, and 1373 unvaccinated SARS-CoV-2-infected individuals without pneumonia. Among the 928 patients tested for autoantibodies against type I IFN, a quarter (234) were positive and were excluded.

**Results:**

No gene reached genome-wide significance. Under a recessive model, the most significant gene with at-risk variants was *TLR7*, with an OR of 27.68 (95%CI 1.5–528.7, *P* = 1.1 × 10^−4^) for biochemically loss-of-function (bLOF) variants. We replicated the enrichment in rare predicted LOF (pLOF) variants at 13 influenza susceptibility loci involved in TLR3-dependent type I IFN immunity (OR = 3.70[95%CI 1.3–8.2], *P* = 2.1 × 10^−4^). This enrichment was further strengthened by (1) adding the recently reported *TYK2* and *TLR7* COVID-19 loci, particularly under a recessive model (OR = 19.65[95%CI 2.1–2635.4], *P* = 3.4 × 10^−3^), and (2) considering as pLOF branchpoint variants with potentially strong impacts on splicing among the 15 loci (OR = 4.40[9%CI 2.3–8.4], *P* = 7.7 × 10^−8^). Finally, the patients with pLOF/bLOF variants at these 15 loci were significantly younger (mean age [SD] = 43.3 [20.3] years) than the other patients (56.0 [17.3] years; *P* = 1.68 × 10^−5^).

**Conclusions:**

Rare variants of TLR3- and TLR7-dependent type I IFN immunity genes can underlie life-threatening COVID-19, particularly with recessive inheritance, in patients under 60 years old.

**Supplementary Information:**

The online version contains supplementary material available at 10.1186/s13073-023-01173-8.

## Background

Clinical variability is high in unvaccinated individuals infected with severe acute respiratory syndrome coronavirus 2 (SARS-CoV-2), ranging from silent infection to lethal disease. In ~ 3% of cases, infection leads to critical COVID-19 pneumonia, requiring high-flow oxygen (O_2_ > 6 L/min), mechanical ventilation (non-invasive or by intubation), or extracorporeal membrane oxygenation (ECMO) [1]. Advanced age is by far the strongest predictor of COVID-19 severity, with the risk of death doubling every 5 years of age from childhood onward [2, 3]. Men are also at greater risk of death than women [3–5]. Genome-wide (GW) association studies have identified several common loci associated with COVID-19 severity, the most significant being a region on chromosome 3p21.31 that was introduced by archaic introgression from Neanderthals [6–10]. The risk haplotype encompasses six genes (*SLC6A20, LZTFL1, CCR9, FYCO1, CXCR6,* and *XCR1*) and confers an estimated OR per copy of between 1.6 and 2.1, with higher values for individuals under 60 years old [7, 11]. Twenty-four GW regions have been shown to be significantly associated with critical COVID-19 [10–12]. Four of these regions encompass genes involved in type I IFN immunity. The first, on chr12q24.13, containing protective variants, is also a Neanderthal haplotype [13] and includes the *OAS1*, *OAS2*, and *OAS3* cluster, these interferon-stimulated genes (ISGs) being required for the activation of antiviral RNaseL. The second, a region on chr21q22.1, includes *IFNAR2*. The third, a region on chr19p13.2, includes *TYK2*. The fourth, a region on chr9p21, includes *IFNA10*. However, common variants have a modest effect size and explain only a very small fraction of the clinical variability [6, 8]. This prompted us to search for rare variants conferring a stronger predisposition to life-threatening COVID-19.

**Fig. 1:**
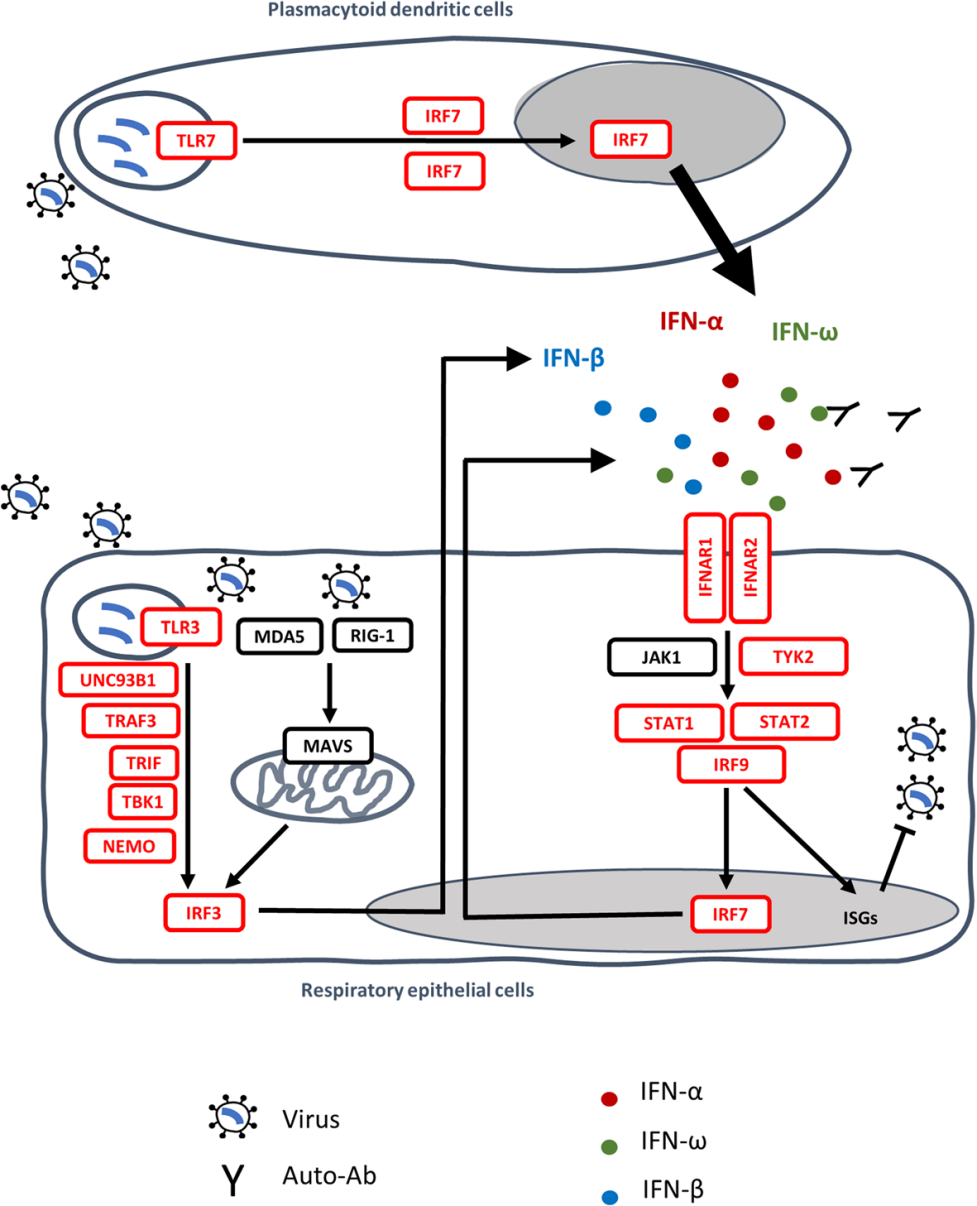
Type I IFN immunity genes associated with life-threatening COVID-19. Inborn errors of type I IFN immunity and autoantibodies neutralizing type I IFNs (*α*, *β*, *ω*) underlie life-threatening COVID-19 pneumonia by interfering with type I IFN immunity in respiratory epithelial cells (RECs) and blood plasmacytoid dendritic cells (pDCs). SARS-CoV-2 infection can induce type I IFN production in a TLR3-dependent manner in tissue-resident RECs (which express TLR3 but not TLR7) and in a TLR7-dependent manner in circulating pDCs (which express TLR7 but not TLR3). IRF7 is constitutively expressed in pDCs, at higher levels than in other cell types, whereas it is mostly induced by viral infection in RECs. Reported in red are the 13 genes (*IFNAR1, IFNAR2, IRF3, IRF7, IRF9, IKBKG, STAT1, STAT2, TBK1, TICAM1, TLR3, TRAF3,* and *UNC93B1*) investigated in a previous study [15]; *TYK2* and *TLR7* were subsequently shown to underlie severe COVID-19 [19, 30]

Through a candidate approach focusing on influenza susceptibility genes, the COVID Human Genetics Effort (CHGE [14]) provided proof-of-concept that autosomal inborn errors of TLR3-dependent and -independent type I interferon (IFN) immunity, including autosomal recessive (AR) deficiencies of IFNAR1 or IRF7, can underlie critical COVID-19 [15]. Other children with AR IFNAR1, IFNAR2, TBK1, or STAT2 deficiency were subsequently reported, as well as children with AR TYK2 deficiency [16–20] (Fig. 1). Some other groups were unable to replicate these findings, but the variants were not tested biochemically and it is unclear whether recessive defects were considered [11, 21–23]. There may also be other reasons for their findings [1, 24], the most important being the age distribution of the case cohorts. The other case cohorts were much older than ours (mean age of 66 vs. 52 years) and we found that inborn errors of immunity (IEI) were more frequent in patients under 60 years old [25]. Consistently, we recently reported that ~ 10% of children with moderate, severe, or critical COVID-19 pneumonia had recessive inborn errors of type I IFN immunity [19]. Moreover, older patients are more likely to carry pre-existing autoantibodies (auto-Abs) neutralizing type I IFN, which are found in about 15% of critical cases and up to 21% of patients over the age of 80 years [26, 27]. The presence of such auto-Abs has been replicated by at least 26 studies worldwide [28, 29], and we also recently showed that autoimmunity to type I IFNs is a strong common predictor of COVID-19 death in unvaccinated individuals, providing further evidence for the role of type I IFN immunity in life-threatening COVID-19 [29].

Using an unbiased X-wide gene burden test, we also identified X-linked recessive (XR) TLR7 deficiency in 17 male patients aged 7–71 years with critical COVID-19 pneumonia, accounting for ~ 1% of cases in men (Fig. 1) [30]. Moreover, six of the 11 *TLR7* variants previously reported in patients from other studies were deleterious (carried by nine of 16 patients) [31–36], whereas the *TLR7* variants in other studies were not disclosed [21, 22]. TLR3 senses viral dsRNA in respiratory epithelial cells, whereas TLR7 senses ssRNA in plasmacytoid dendritic cells [25, 28]. Both pathways induce the production of type I IFNs. *TLR7* gain-of-function variants were recently shown to be associated with human systemic lupus erythematosus [37], providing an example of mirror genetic effects between infectious and inflammatory/autoimmune diseases [38]. Collectively, these findings suggest that type I IFNs are essential for protective immunity to SARS-CoV-2 in the respiratory tract, with insufficient type I IFN activity accounting for up to 15–20% of cases of life-threatening COVID-19. Despite this high proportion, the determinants of critical COVID-19 pneumonia remain to be identified in ~ 80% of cases. Here, we tested the hypotheses that other IEI may underlie critical COVID-19 pneumonia in at least some patients and that our initial findings could be replicated in a new cohort. With the CHGE, we performed a GW gene-based rare variant association analysis. This analysis was performed in both previously investigated patients who had not been screened at the GW level [15, 19, 30], and in newly recruited patients. We also tested the hypothesis that we could replicate our initial finding of an enrichment in pLOF variants of candidate type I IFN-related genes in newly recruited patients, given the controversy from other groups. We extended the analysis to two other type I IFN-related genes, *TLR7* and *TYK2*, that we had recently found to be associated with critical COVID-19 [19, 30], and to branchpoint (BP) variants with a potentially strong impact on the splicing of the 15 type I IFN-related genes [39]. Finally, we refined the analysis of the type I IFN-related genes by taking age, sex, and zygosity into account.

## Methods

### Cohort

Since the beginning of the pandemic, we have enrolled more than 9000 individuals with SARS-CoV-2 infection and broad clinical manifestations from all over the world through the COVID Human Genetic Effort (CHGE). In this study, we focused on 3503 patients with life-threatening COVID-19 and 1373 individuals with asymptomatic/mild infection. Life-threatening COVID-19 cases were defined as patients with pneumonia who developed critical disease, whether pulmonary with high-flow oxygen (> 6 L/min) or mechanical ventilation [continuous positive airway pressure (CPAP), bilevel positive airway pressure (BIPAP), and intubation], septic shock, or any other type of organ damage requiring intensive care unit admission. We screened for the presence of autoantibodies (auto-Abs) against type I IFNs in all patients for whom plasma was available (*N* = 928), as previously described [26, 27], and we excluded 234 patients who tested positive for auto-Abs as they already have a major risk factor for developing critical COVID-19 [29]. In total, 3269 patients with life-threatening COVID-19 were included in the analysis. Among those 3269 patients, 1301 had been included in previous studies restricted to a short list of 18 candidate genes [15, 19] or to the X chromosome [30], and 1968 had not been studied before. Controls were defined as individuals infected with SARS-CoV-2 who remained asymptomatic or pauci-symptomatic, with the presence of mild, self-healing, ambulatory disease (*N* = 1373). The presence of infection was assessed on the basis of a positive PCR test and/or serological test and/or the presence of typical symptoms such as anosmia or agueusia after exposure to a confirmed COVID-19 case. Whole-exome (*N* = 2003 cases and 866 controls) or whole-genome (*N* = 1266 cases and 507 controls) sequencing was performed for the cases and controls, and high-quality variants were obtained from the sequencing data as detailed in the Additional file 1: Supplementary Methods.

### Population stratification

Principal component analysis (PCA) was performed with PLINK v1.9 software [40] on a pruned subset of ~ 14,600 SNPs not in linkage disequilibrium (maximum r2 value for linkage disequilibrium 0.4 between pairs of SNPs) with a minor allele frequency (MAF) > 1%, call rate > 99%, and *P* value for departure from Hardy–Weinberg equilibrium > 10^−5^, as previously described [41]. Ethnic origin was inferred from the PCA as previously described [41].

### Variant selection

For each gene, we considered several sets of candidate coding variants, defined according to (i) functional annotation: predicted loss-of-function (pLOF) variants only (including stop gain/lost, start lost, frameshift, or splice variants), or pLOF with missense and in-frame variants (MISSLOF); (ii) the gnomAD v2.1 allele frequency (AF): variants with a gnomAD allele frequency below 1%, 0.1%, or 0.01%; and (iii) Combined Annotation Dependent Depletion (CADD) score [42] for missense and in-frame variants: CADD score ≥ mutation significance cut-off (MSC) for the corresponding gene [43] or all variants regardless of the CADD score. We considered nine sets of variants in total: (1) pLOF variants with gnomAD AF < 1%; (2) pLOF variants with gnomAD AF < 0.1%; (3) pLOF variants with gnomAD AF < 0.01%; (4) MISSLOF with CADD > MSC and gnomAD AF < 1%; (5) MISSLOF with CADD > MSC and gnomAD AF < 0.1%; (6) MISSLOF with CADD > MSC and gnomAD AF < 0.01%; (7) MISSLOF with gnomAD AF < 1%; (8) MISSLOF with gnomAD AF < 0.1%; (9) MISSLOF with gnomAD AF < 0.01%.

### Rare variant burden analysis

We performed a genome-wide gene-based rare variants burden analysis. For each gene, the genotypic information for candidate rare variants was summarized into a genetic score defined according to three genetic models: (1) co-dominant: samples were coded 2 if they carried at least one biallelic variant, 1 if they carried at least one monoallelic variant, and 0 otherwise; (2) heterozygous: samples were coded 1 if they carried at least one monoallelic variant and 0 otherwise; and (3) recessive: samples were coded 1 if they carried at least one biallelic variant and 0 otherwise. For the X chromosome, hemizygous males are considered to be equivalent to homozygous females. The association between the genetic score for each gene and the disease status was assessed with a logistic regression-based likelihood ratio test (LRT) from EPACTS (Efficient and Parallelizable Association Container Toolbox) [44] for the genome-wide burden analysis or R 3.6.0 [45] for the candidate type I IFN-related pathway. Firth’s bias correction, with the fast.logistf.fit function of EPACTS or the logistf function of the R logistf package [46], was applied if the *P* value of the LRT was below 0.05. Analyses were adjusted for sex, age (in years), and the first five PCs of the PCA In Firth’s regression, a penalty term is assigned to the standard maximum likelihood function used to estimate the parameters of a logistic regression model when there are rare events or when complete separation exists [47]. With no covariates, this corresponds to adding 0.5 to every cell of a 2 by 2 table of allele counts versus case–control status. For a given gene and variant set, the burden test was not performed if the number of carriers across all samples was below 3.

We used three analysis strategies: (1) joint analysis of all samples; (2) trans-ethnic meta-analysis: the analysis was stratified according to 7 inferred ancestry subgroups (African, North African, European, admixed American, Middle Eastern, South Asian, East Asian). For each subgroup, an ethnicity specific PCA was performed and used in the logistic regression model; and (3) trans-pipeline meta-analysis to account for heterogeneity due to the type of sequencing data: the analysis was stratified according to the type of data shared (FASTQ vs. VCF). Subgroup *P* values were subjected to further meta-analysis, accounting for the direction of the effect and sample size, with METAL [48].

### Correction for multiple testing

For each gene, up to 9 burden tests were performed per genetic model. These tests were not independent; we therefore assessed the effective number of burden tests Meff with a method adapted from that described by Patin et al. [49], based on the approach of Li and Ji [50]. This approach makes use of the variance of the eigenvalues of the observed statistics correlation matrix to estimate Meff. The Bonferroni-corrected threshold was then defined as 0.05/Meff.

### Odds ratio (OR) equality for homozygous/hemizygous versus heterozygous carriers of pLOF variants at type I IFN genes

We investigated whether the odds of critical COVID-19 differed for carriers and non-carriers of pLOF variants at the type I IFN immunity loci as a function of zygosity (homozygous/hemizygous vs heterozygous). In the full sample, we used LRT to compare a full Firth bias-corrected logistic regression model including two different parameters for carriers of pLOF as a function of zygosity (alternative hypothesis) with a Firth bias-corrected logistic regression model including only one parameter for carriers of pLOF, not taking zygosity into account (null hypothesis). The analysis was performed with the R logistf package.

### Biochemical characterization of TLR7 variants with a luciferase reporter assay

We tested the *TLR7* variants as previously described [30]. Briefly, TLR7 variants were generated by site-directed mutagenesis. The WT or variant alleles were re-introduced into a Myc-DDK-pCMV6 vector (Origene). HEK293T cells, which have no endogenous TLR7 expression, were transfected with 50 ng of Myc-DDK-pCMV6 vector, empty or containing the WT or a variant allele the reporter construct pGL4.32 (100 ng), and an expression vector for Renilla luciferase (10 ng), with the X-tremeGENE™ 9 DNA Transfection Reagent kit (Sigma-Aldrich). The pGL4.32 (luc2P/NF-κB–RE/Hygo) (Promega) reporter vector contains five copies of the NF-κB–responsive element (NF-κB–RE) linked to the luc2P luciferase reporter gene. After 24 h, the transfected cells were left unstimulated or were stimulated with R848 (1 μg/ml; resquimod), for activation via TLR7/8 (Invivogen), or R837 (5 μg/ml; imiquimod) (Invivogen), or CL264 (5 μg/ml; Invivogen), human TLR7-specific agonists, for 24 h. Relative luciferase activity was then determined by normalizing the values obtained against the firefly:Renilla luciferase signal ratio.

## Results

### Cohort description

**Fig. 2:**
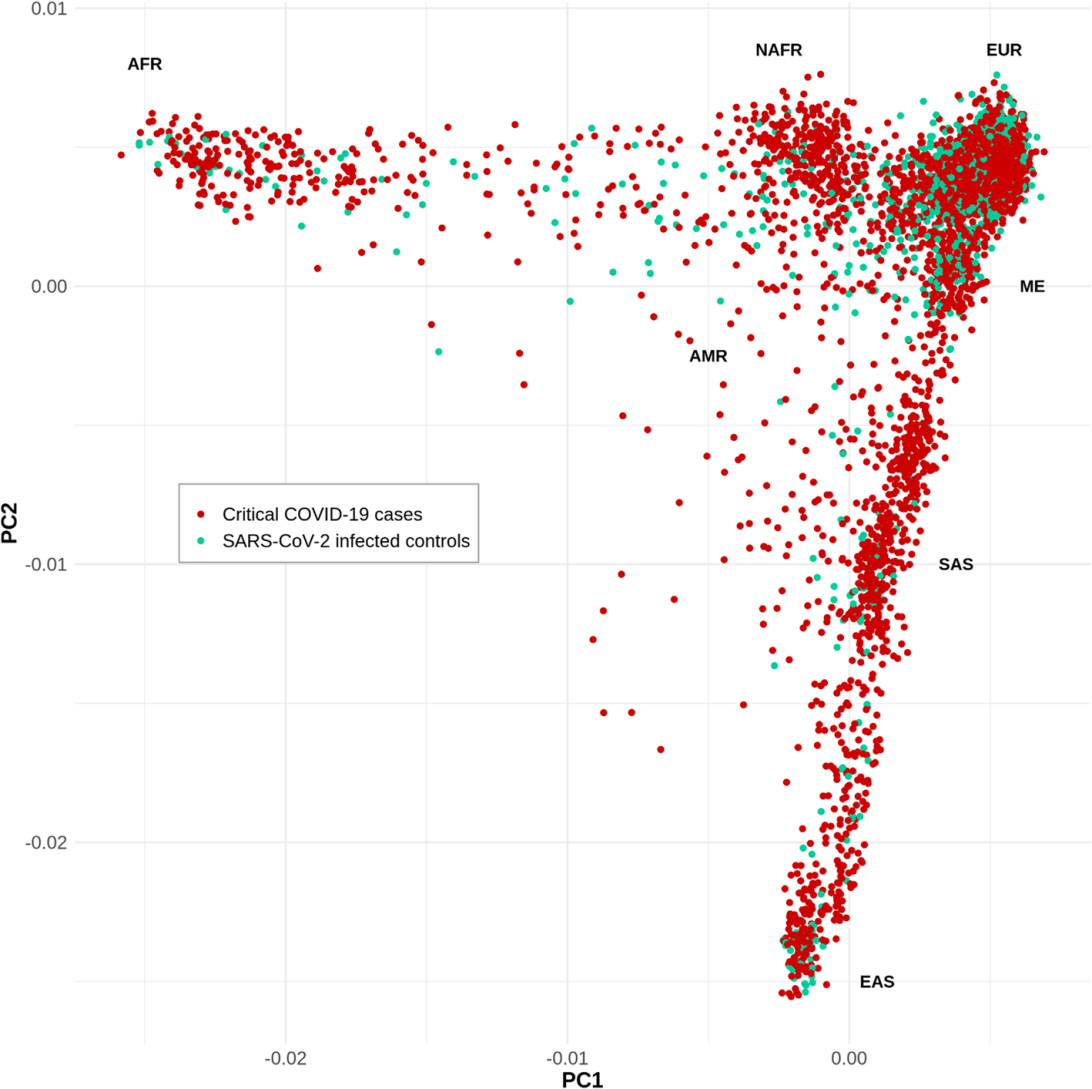
Principal component analysis of patients with life-threatening COVID-19 (red) and SARS-CoV-2-infected controls (green). Principal component analysis (PCA) was performed with PLINK v1.9 software [40] on a pruned subset of ~ 14,600 exonic SNPs in linkage equilibrium (maximum *r*^2^ value for linkage disequilibrium of 0.4 between pairs of SNPs) with a minor allele frequency (MAF) > 1%, call rate > 99% and *P* value for departure from Hardy–Weinberg equilibrium > 10.^−5^. Samples were of diverse ethnic origins, including European (EUR), admixed American (AMR), North African (NAFR), sub-Saharan African (AFR), Middle Eastern (ME), South Asian (SAS), and East Asian (EAS)

Through the CHGE, we collected whole-exome sequencing (WES) or whole-genome sequencing (WGS) data for 3503 patients with life-threatening COVID-19 pneumonia (hereafter referred to as “patients”; see Supplemental Methods) and 1373 individuals with mild or asymptomatic infection, i.e., without pneumonia (hereafter referred to as “controls”). In total, 928 of the 3503 patients were screened for the presence of auto-Abs against type I IFN [26, 27] (Supplemental Methods) and the 234 patients who tested positive were excluded from this analysis as they already have a major risk factor for the development of critical COVID-19 [29]. In total, 1301 of the 3269 remaining patients had been included in previous studies restricted to a short list of 18 candidate genes [15, 19] or to the X chromosome [30], and 1968 had not been studied before. The mean age (SD) of the patients was 55.7 (17.4) years, with a male-to-female ratio of 2.4 (Table 1). The controls were significantly younger than the patients (*P* < 0.0001), with a mean age (SD) of 43.8 years (20.1 years) and were more likely to be female (*P* < 0.0001; male-to-female ratio = 0.7). The patients and controls were of various ethnic origins, mostly of European and Middle Eastern ancestry, according to principal component analysis (PCA) (Fig. 2). Raw sequencing data were either centralized in the HGID laboratory and processed with the HGID pipeline (2492 cases and 870 controls) or processed separately by each sequencing hub (777 cases and 503 controls; See Supplemental Methods). A joint analysis was performed first on the combined sample of 3269 patients and 1373 controls. Given the heterogeneity of the cohort due to different ancestries and processing pipelines, we also performed a trans-ethnic and a trans-pipeline meta-analysis; only results consistent across the three analyses are reported here (See Supplemental Methods).

**Table 1:** Baseline characteristics of study participants

	**Life-threatening COVID-19**	**Infected controls**	***P* value**^**a**^
	***n* = *3269***	***n* = *1373***	
**Sex**, no. (%)			< 0.0001
Male	2314 (70.8%)	542 (39.5%)	
Female	955 (29.2%)	831 (60.5%)	
**Age (years)**			
Mean (SD)	55.74 (17.40)	43.83 (20.14)	< 0.0001
Median (range)	57 (0.08–99)	43 (0.08–105)	
**Processing pipeline,** no. (%)			< 0.0001
HGID laboratory	2492 (76.2%)	870 (63.4%)	
Other	777 (24.8%)	503 (36.6%)	
**Ancestry,** no. (%)			< 0.0001
European	1374 (42.0%)	960 (69.9%)	
Middle Eastern	483 (14.8%)	158 (11.5%)	
Admixed American	466 (14.3%)	109 (7.9%)	
North African	300 (9.2%)	24 (1.7%)	
South Asian	279 (8.5%)	36 (2.6%)	
Sub-Saharan African	234 (7.1%)	43 (3.1%)	
East Asian	133 (4.1%)	43 (3.1%)	

*HGID* Human Genetics of Infectious Diseases

^a^Chi-squared tests were used to compare proportions, and *t* tests were used to compare the mean ages

### Genome-wide analysis under a co-dominant model

**Fig. 3:**
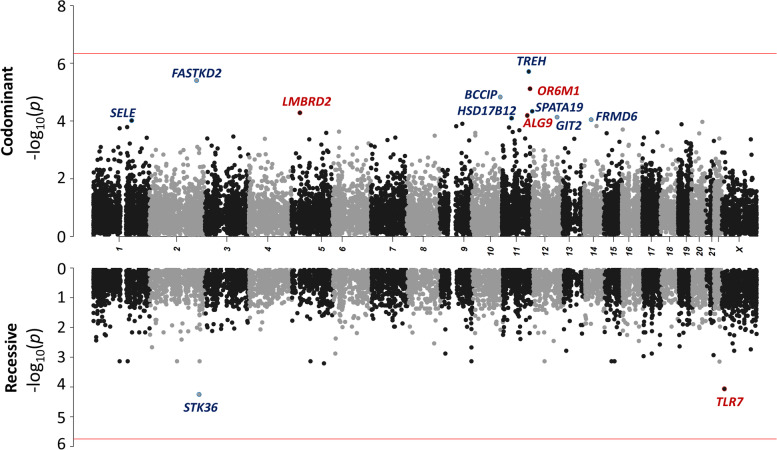
Manhattan plot for genome-wide burden analysis under the co-dominant (top) and recessive (bottom) models. For each gene, the negative log-transformed *p* value of the joint analysis for the most significant variant set under a co-dominant (top) or recessive (bottom) model is plotted. For each gene, variant sets providing inconsistent results across the joint analysis, the trans-ethnic meta-analysis, and the trans-pipeline meta-analysis (i.e., *P* < 0.001 in the joint analysis and *P* > 0.05 in the trans-ethnic or trans-pipeline meta-analysis) were discarded. The red lines represent the significance threshold after Bonferroni correction to account for the total number of independent tests (*P* = 4.61 × 10^−7^ under a co-dominant model and 1.85 × 10^−6^ under a recessive model). The names of the top-ranked genes with a joint *P* < 10^−4^ are shown in red for rare variants associated with an increase in the risk of critical COVID-19 and in blue otherwise

We first performed a GW rare variant burden analysis on the 3269 patients with life-threatening COVID-19 and 1373 controls with asymptomatic/mild COVID-19 under a co-dominant model, using nine sets of variants (See Supplemental Methods). The QQ plots for the joint analysis of the samples revealed no systematic deviations from the null hypothesis, and the genomic inflation factors (*λ*) were close to 1 (Additional file 2: Table S1). In total, 18,064 genes were analyzed with at least one of the nine variant sets, resulting in an effective number of independent tests (Meff) for the joint analysis of 108,384, giving a Bonferroni-corrected significance threshold of 4.61 × 10^−7^. No gene was found to be of GW significance (see the Manhattan plot in Fig. 3A, Additional file 2: Table S2). The gene with the strongest association was *TREH,* encoding the trehalase enzyme, which hydrolyses trehalose, with rare (gnomAD allele frequency [AF] < 10^−4^) nonsynonymous variants associated with a lower risk of life-threatening COVID-19 (OR = 0.12[95% CI 0.05–0.28], *P* = 1.9 × 10^−6^; Additional file 2: Table S3). In analyses of genes for which rare predicted loss-of-function (pLOF) variants were associated with an increase in the risk of life-threatening COVID-19 (Table 2), the strongest association was that for *NPC2*, for rare (gnomAD AF < 0.01) pLOF variants, with 28 heterozygous carriers among patients (0.9%), and four heterozygous carriers (0.3%) among controls (OR = 5.41 [95% CI 1.8–16.4], *P* = 5.8 × 10^−4^). *NPC2* encodes the Niemann-Pick disease type C2 protein and homozygous LOF mutations of this gene cause Niemann-Pick disease [51]. NPC2 interacts with NPC1, which is also an essential endosomal receptor for the Ebola virus [52, 53]. Both NPC1 and NPC2 were implicated in the regulation of SARS-CoV-2 entry in a CRISPR screen [54]. The GW burden analysis under a dominant model yielded similar conclusions (Additional file 2: Table S3).

**Table 2:** Top results of the genome-wide burden analysis for rare pLOF variants increasing the risk of life-threatening COVID-19 under a co-dominant model

**Chr**	**Gene**	**GnomAD AF threshold**	**No. carriers of at least one (no. homozygous) pLOF variant**		**Joint analysis**		**Trans-ethnic meta-analysis**	**Trans-pipeline meta-analysis**
			**Cases** ***(n* = *3269)***	**Controls** ***(n* = *1373)***	**OR[95%CI]**	***P* value**	***P* value**	***P* value**
14	*NPC2*	0.01	28 (0)	4 (0)	5.41[1.8–16.4]	5.8 × 10^−4^	2.1 × 10^−3^	3.3 × 10^−4^
3	*DLEC1*	0.01	56 (0)	16 (0)	2.55[1.3–4.9]	3.6 × 10^−3^	0.013	4.9 × 10^−3^
13	*NEK5*	0.001	16 (0)	0 (0)	27.03[0.9–864.2]	4.0 × 10^−3^	1.5 × 10^−3^	0.011
5	*CCNI2*	0.01	19 (1)	1 (0)	7.15[1.2–43.1]	4.1 × 10^−3^	4.1 × 10^−3^	5.0 × 10^−3^
22	*C22orf29*	0.001	13 (0)	0 (0)	15.6[0.8–315.8]	4.5 × 10^−3^	7.9 × 10^−3^	4.6 × 10^−3^
20	*DLGAP4*	0.001	37 (0)	3 (0)	4.35[1.3–14.5]	4.8 × 10^−3^	8.3 × 10^−3^	0.011

Only genes with a *P* value ≤ 5 × 10^−3^ in the joint analysis and *P* values < 0.05 in trans-ethnic and trans-pipeline meta-analyses are displayed

*AF* allele frequency

### Genome-wide analysis under a recessive model

We then performed a GW screen under a recessive model (autosomal and X-linked). In total, 4511 genes were analyzed with at least one of the nine variant sets, resulting in 27,066 independent tests, giving a Bonferroni-corrected significance threshold of 1.85 × 10^−6^. No gene reached GW significance (Fig. 3B). In analyses of genes with rare variants increasing the risk of life-threatening COVID-19, *TLR7* was, by two orders of magnitude, the most significant gene, with 51 carriers (1.6%) of at least one rare (gnomAD AF < 0.01) missense or pLOF variant in patients versus two carriers (0.1%) in controls (OR = 8.41[95% CI 1.9–35.5], *P* = 8.95 × 10^−5^) (Table 3). Most of the carriers were male, with only one carrier among the patients and one among the controls being female. The variants carried by the two controls were previously shown to be biochemically neutral [19, 30] (Additional file 2: Table S4). The 51 cases carried 33 different variants, 13 of which had been shown to be neutral; 16 were previously shown to be hypomorphic or amorphic [19, 30], and four were previously unknown. The four new variants were tested: one was found to be neutral and the other three were deleterious (Additional file 1: Fig S1). Restricting the analysis to biochemically proven LOF variants (bLOF) decreased the number of carriers (20 cases vs. 0 controls), but the association signal remained highly significant, with a much higher odds ratio (OR = 27.68 [95% CI 1.5–528.7], *P* = 1.08 × 10^−4^) (Table 3). These findings confirm that *TLR7* is a critical COVID-19 susceptibility locus, responsible for 0.9% of critical cases in male patients.

**Table 3:** Top results of the genome-wide burden analysis for rare variants increasing the risk of life-threatening COVID-19 under a recessive model

**Chr**	**Gene**	**Variant set**	**Type of variant**	**CADD > MSC**^**a**^	**GnomAD AF threshold**	**No. carriers of at least one rare homo-/hemizygous variant**		**Joint analysis**		**Trans-ethnic meta-analysis**	**Trans-pipeline meta-analysis**
						**Cases *(n* = *3269)***		**Controls *(n* = *1373)***		**OR[95%CI]**	***P* value**
***GW analysis***											
X	*TLR7*	7	MISSLOF	FALSE	0.01	51	2	8.41 [1.9–35.5]	8.95 × 10^−5^	7.04 × 10^−4^	2.66 × 10^−4^
14	*AHNAK2*	5	MISSLOF	TRUE	0.001	37	2	4.45 [1.1–17.7]	0.01	2.15 × 10^−3^	8.84 × 10^−3^
***Refined analysis on TLR7***											
X	*TLR7*		bLOF	-	0.01	20	0	27.68[1.5–528.7]	1.1 × 10^−4^	6.6 × 10^−3^	2.7 × 10^−4^

Only genes with *P* values ≤ 0.01 in the joint analysis and *P* values < 0.05 in trans-ethnic and trans-pipeline meta-analyses are displayed

*AF* allele frequency

^a^Combined Annotation Dependent Depletion (CADD) score [42] greater than the Mutation Significance Cut-off (MSC) for the corresponding gene. The MSC is defined for a given gene as the lower limit of the confidence interval (95%) of the CADD score of all its known pathogenic mutations [43]

### Genome-wide gene-based analysis including common variants

Published GWAS identified a number of common variants associated with severe COVID-19 pneumonia [8, 10, 12, 55]. We then assessed the combined effect of common and rare candidate coding variants at the gene level, in a weighted burden approach [56], as detailed in the Supplemental Methods. Briefly, for each individual, we calculated a genetic score by summing the number of minor alleles for each variant and weighting this sum by the frequency of the variant [57]. We then tested the association between this genetic score and case–control status in a logistic regression framework. As described above, we focused on pLOF only, pLOF and in-frame variants with CADD > MSC, or pLOF and in-frame variants without filtering on CADD score (Additional file 2: Table S5). As in the analysis focusing on rare variants only, no gene reached genome-wide significance after correction for multiple testing in this analysis considering both rare and common variants. The top-ranked gene, with consistent results across the joint analysis and the trans-ethnic and trans-pipeline meta-analyses, was *TREH*, with a protective effect against life-threatening COVID-19 of pLOF or nonsynonymous variants with a CADD score greater than the MSC (OR = 0.85 [95%CI 0.78–0.91], *P* = 3.6 × 10^−6^). Finally, we analyzed 20 candidate genes identified by GWAS for critical pneumonia in more detail [8, 10, 12, 55]. No significant association was detected for any of these genes (Additional file 2: Table S6), even with a relaxed Bonferroni threshold of 2.5 × 10^−3^, accounting for the number of GWAS genes.

### Enrichment in rare pLOF variants at 13 type I IFN-related influenza susceptibility loci

Following on from our initial analysis [15], we also performed a candidate pathway enrichment analysis focusing on the 13 genes involved in Toll-like receptor 3 (TLR3)– and interferon regulatory factor 7 (IRF7)–dependent type I IFN immunity to influenza virus (*IFNAR1, IFNAR2, IRF3, IRF7, IRF9, IKBKG, STAT1, STAT2, TBK1, TICAM1, TLR3, TRAF3,* and *UNC93B1*) (Fig. 1). We confirmed the significant enrichment in rare (gnomAD AF < 10^−3^) pLOF variants at the 13 loci in patients with critical COVID-19, with 34 carriers among patients versus six among controls (OR = 3.70 [95% CI 1.7–9.5], *P* = 2.1 × 10^−4^ under a co-dominant model; Table 4). We also estimated this *p* value in a simulation study taking 13 loci randomly selected from a set of genes with similar pLI and CoNeS values (see Additional file 1: Supplemental Methods); we obtained an empirical *p* value of 3.7 × 10^−4^. Our cohort included 551 patients and 314 controls already screened for pLOF variants of the 13 genes included in our previous study [15] (Additional file 2: Table S7). The exclusion of these 551 cases and 314 controls resulted in a similar conclusion of enrichment in rare pLOF at the 13 loci (OR = 3.21 [95% CI 1.3–8.2], *P* = 5.97 × 10^−3^) formally replicating our initial association. Significant replication was also observed in the trans-ethnic (*P* = 0.01) and the trans-pipeline (*P* = 0.009) analyses. We found that 31 of the 34 carriers of pLOF variants were heterozygous, and three were homozygous: one for a frameshift variant of *IRF7* described in a previous study [15], one for a previously reported deletion spanning 4394 base pairs in *IFNAR1* [16, 19], and one for a previously unknown deletion spanning 6624 base pairs of *IFNAR1* (Additional file 2: Table S8). All the biallelic pLOF variants were found in patients. Consequently, the OR for homozygous carriers (OR = 15.79 [95%CI 1.4–2170.4], *P* = 0.02) was higher than that for heterozygous carriers (OR = 3.11 [95%CI 1.4–8.6], *P* = 5.2 × 10^−3^), but both were significant.

**Table 4:** Enrichment analysis of rare pLOF/bLOF variants in genes involved in type I IFN immunity

**Gene set**	**Cohort**	**Model**	**No. carriers**		**Joint analysis**		**Trans-pipeline meta-analysis**	**Trans-ethnic meta-analysis**
			**Cases**	**Controls**	***P* value**	**OR[95%CI]**	***P* value**	***P* value**
13 genes^a^	Samples independent of [15]^b^	Co-dominant	25	5	5.97 × 10^−3^	3.21 [1.3–8.2]	9.15 × 10^−3^	0.01
13 genes	Full^c^	Co-dominant	34	6	2.13 × 10^−4^	3.70 [1.7–9.5]	7.45 × 10^−4^	6.52 × 10^−4^
13 genes	Full	Heterozygous only^d^	31	6	5.21 × 10^−3^	3.11 [1.3–8.6]	7.88 × 10^−3^	5.98 × 10^−3^
13 genes	Full	Recessive	3	0	0.02	15.79 [1.4–2170.4]	0.05	0.03
13 genes + *TYK2*	Full	Co-dominant	37	7	1.40 × 10^−4^	3.30 [1.6–7.8]	5.77 × 10^−4^	5.64 × 10^−4^
13 genes + *TYK2*	Full	Heterozygous only	32	7	0.02	2.53 [1.1–6.6]	0.03	0.02
13 genes + *TYK2*	Full	Recessive	5	0	3.36 × 10^−3^	19.65 [2.1–2635.4]	9.84 × 10^−3^	0.03
13 genes + *TYK2* + bLOF *TLR7*	Full	Co-dominant	57	9	1.27 × 10^−7^	3.82 [2.0–7.2]	1.99 × 10^−7^	2.20 × 10^−6^
13genes + *TYK2* + bLOF *TLR7*	Full	Heterozygous only	32	9	0.04	2.27 [1.0–5.2]	0.04	0.02
13genes + *TYK2* + bLOF *TLR7*	Full	Recessive	25	0	4.69 × 10^−7^	39.19 [5.2–5037.01]	2.39 × 10^−6^	6.66 × 10^−5^
13genes + *TYK2* + bLOF *TLR7* + BP variants	Full	Co-dominant	67	9	7.7 × 10^−8^	4.40 [2.3–8.4]	3.5 × 10^−8^	6.5 × 10^−7^

^a^*IFNAR1, IFNAR2, IRF3, IRF7, IRF9, IKBKG, STAT1, STAT2, TBK1, TICAM1, TLR3, TRAF3* and* UNC93B1*

^b^2718 patients and 1059 controls newly recruited and not screened in [15]

^c^The full cohort includes 3269 patients and 1373 controls

^d^In this model, only subjects with heterozygous variants are considered as carriers

### Inclusion of TYK2 and TLR7 genes and branchpoint variants

Since the publication of the aforementioned study [15], AR TYK2 deficiency has been reported in children with COVID-19 pneumonia [19]. We identified two patients homozygous for a rare pLOF variant of *TYK2* already described in a previous study [19] and one patient and one control heterozygous for a rare pLOF variant (Additional file 2: Table S8). Adding these patients to the analysis gave very similar results under a co-dominant model (OR = 3.30[95% CI 1.6–7.8], *P* = 1.4 × 10^−4^) and strengthened the evidence for association under a recessive model (OR = 19.65[95% CI 2.1–2635.4], *P* = 3.4 × 10^−3^) (Table 4). An analysis of the rare pLOF variants at these 14 loci plus the bLOF variants of *TLR7* revealed highly significant enrichment (OR = 3.82 [95%CI 2.0–7.2], *P* = 1.3 × 10^−7^ under a co-dominant model). The effect was stronger for homozygous/hemizygous carriers (OR = 39.19 [95%CI 5.2–5037.01], *P* = 4.7 × 10^−7^) than for heterozygous carriers (OR = 2.27 [95%CI 1.0–5.2], *P* = 0.04), and these two ORs were significantly different (*P* = 0.008). We further screened the full cohort of cases and controls for intronic branchpoint (BP) variants, which might potentially have a strong impact on splicing and be considered pLOF variants, in the 15 type I IFN-related genes, with our new tool BPHunter [39]. We identified six branchpoint (BP) variants (Additional file 2: Table S9) carried in heterozygous state by 10 additional cases and no controls. Adding these BP variants to the analysis of the 15 type I IFN-related loci under a co-dominant model further strengthened the association signal (OR = 4.40 [2.3–8.4], *P* = 7.7 × 10^−8^) (Table 4).

### Age and sex stratified analysis of the 15 type I IFN-related loci

Advanced age is the strongest risk factor for life-threatening COVID-19. Male individuals are also at higher risk than female individuals. As for the main GWAS hits [58, 59], we performed an analysis stratified for age and sex for the 15 type I IFN-related loci. The analysis stratified for sex revealed a much stronger association signal in male than in female individuals, as expected given the X-linked recessive mode of inheritance of TLR7 deficiency (Additional file 2: Table S10). Nevertheless, the enrichment in rare pLOF variants at the 15 loci in female subjects remained significant under a co-dominant model (*P* = 0.02) and a recessive model (*P* = 0.05). The addition of the BP variants strengthened the association signal in female subjects under a co-dominant model (*P* = 3.7 × 10^−3^). In the analysis stratified for age, we assigned the cases to two age groups (under 60 years of age vs. 60 years and over), which we compared with all controls. We used an age cut-off of 60 years, which was close to the median age of the cases, in accordance with the analyses performed in [7, 59]. The age stratified analysis revealed a strong impact of age, the genetic effect being restricted to younger cases (OR = 4.65 [2.4–9.0], *P* = 2.2 × 10^−9^, Additional file 2: Table S10). Accordingly, the 67 patients with critical COVID-19 carrying a rare pLOF or bLOF variant of one of the 15 genes were significantly younger than the remaining 3202 patients in the cohort (mean age [SD] in years: 43.68 [19.4] vs. 56.0 [17.3] years; *P* = 2.3 × 10^−6^), consistent with our previous reports that IEIs conferring a predisposition to life-threatening COVID-19 are more frequent in young patients [1, 15, 30]. Moreover, the homozygous/hemizygous carriers were significantly younger than the heterozygous carriers (35.2 [20.3] vs. 48.7 [17.1] years, *P* = 0.008, Additional file 1: Fig S2). Overall, these results clearly demonstrate that the search for additional rare variants conferring a strong predisposition to life-threatening COVID-19 benefits from focus on younger patients.

### In-frame nonsynonymous variants at the 15 loci

We further screened our cohort for rare in-frame nonsynonymous variants with a gnomAD AF < 10^−3^ at these type I IFN-related susceptibility loci. For the 13 initial loci, the enrichment disappeared when in-frame nonsynonymous variants were added to pLOF variants under a co-dominant model (OR = 1.08 [95%CI 0.9–1.3], *P* = 0.42) (Additional file 2: Table S11), whereas a non-significant trend persisted under the recessive model (OR = 5.02 [95% CI 0.7–52.7], *P* = 0.06). Focusing exclusively on in-frame variants decreased the strength of this trend considerably, with only eight homozygous carriers among patients and one among controls (OR = 1.14 [0.2–912.5], *P* = 0.68). Adding *TYK2* variants led to similar conclusions (Additional file 2: Table S11). We then added *TLR7* variants and considered the 15 loci together. Under a co-dominant model, the enrichment became non-significant when in-frame nonsynonymous variants were added (OR = 1.15 [1.0–1.4], *P* = 0.09), but enrichment remained significant under a recessive model (OR = 6.54 [2.4–24.8], *P* = 5.3 × 10^−6^; Additional file 2: Table S11). In analyses considering only rare in-frame homozygous/hemizygous nonsynonymous variants, the effect size was smaller, but the enrichment remained significant (OR = 3.52 [1.3–13.3], *P* = 2.8 × 10^−3^). In total, 41 patients carried a rare homozygous/hemizygous in-frame nonsynonymous variant at one of the 15 loci, and 16 of these variants (carried by 16 patients) were *TLR7* in-frame variants already shown to be bLOF. After excluding the *TLR7* bLOF variants, there was no residual significant enrichment in rare in-frame nonsynonymous variants in patients relative to controls, whatever the genetic model considered.

## Discussion

In this exome-wide gene burden analysis for rare variants underlying critical COVID-19, no gene reached GW statistical significance after accounting for multiple testing. We used simulations to determine the power of our sample to detect an association at the 2.5 × 10^−6^ exome-wide significance threshold (Additional file 1: Fig S3); our sample had a power of more than 80% for detecting alleles with a carrier frequency of 5 × 10^−3^ in the general population and a relative risk of critical COVID-19 of at least 6. These results are consistent with those of two previous large exome-wide studies including more than 1000 critical cases and thousands of population-based controls that found no statistically significant autosomal gene burden associations at stringent significance thresholds accounting for the number of phenotypes and variant sets analyzed [11, 21]. However, under a recessive model, the strongest association—albeit not statistically significant at GW level—was obtained with the X-linked *TLR7* gene, for which association has consistently been reported across studies [21, 22, 30, 32], reaching the less conservative exome-wide significance threshold of 2.5 × 10^−6^ in some of these previous studies [21, 22]. It should be stressed that stringent correction for multiple testing, while necessary to avoid false positives, is a conservative strategy, and that the lack of formal statistical significance at a GW level does not preclude biological causality and medical significance. The burden of proof can be provided experimentally via biochemical, virological, and immunological experiments, as our previous studies of *TLR7* in which we showed that biochemically deleterious TLR7 variants blunted the pDC-dependent sensing of SARS-CoV-2 and induction of type I IFN, thereby accounting for ~ 1% of critical pneumonia cases in men [30]. Additional genes may be found by restricting the association analysis to variants experimentally proven to be deleterious.

This analysis also confirms our previous findings of an enrichment in rare pLOF variants of 13 genes involved in TLR3- and IRF7-dependent type I IFN immunity to seasonal influenza virus in critical cases relative to controls with mild/asymptomatic infection [15]. These results were strengthened by the addition of *TYK2*, which was recently shown to underlie severe COVID-19 [19, 20], and *TLR7*, especially under a recessive model. We found that homozygous/hemizygous carriers of rare pLOF or bLOF variants at the 15 loci had a significantly higher risk of life-threatening COVID-19 than heterozygotes. This is consistent with the generally higher clinical penetrance of recessive than dominant IEI [1]. Overall, 1.7% of the patients with life-threatening COVID-19 carried a rare pLOF or bLOF variant at one of the 15 loci, these variants being homozygous/hemizygous in 0.8% of cases. Adding the BP variants at the 15 loci increased the proportion of carriers among patients with life-threatening COVID-19 to 2.1%. One of the *STAT2* BP variants identified (2:56749159:T > A) has already been validated experimentally [39], but the effects of the five other BP variants identified require confirmation. The study of in-frame nonsynonymous variants might also increase this proportion, but would require the experimental characterization of all these variants. Indeed, in analyses restricted to rare in-frame nonsynonymous variants, we detected no significant enrichment in patients relative to controls. This result is not surprising, as we showed in a previous study [15] that less than 15% of the rare in-frame nonsynonymous variants at the 13 loci carried by cases initially studied were bLOF variants, whereas all the pLOF variants were found to be bLOF. Similar results were obtained for *TLR7*, with only 10 of 108 (9.2%) in-frame nonsynonymous variants observed in gnomAD being bLOF [30]. This high proportion of neutral variants strongly affects the power of burden tests and highlights the need for the experimental characterization of variants.

We also showed that patients carrying rare pLOF or bLOF variants of these 15 type I IFN-related genes were significantly younger than the remaining patients (mean age [SD] in years: 43.3 [20.3] vs. 56.0 [17.3] years). This was particularly true for homozygous/hemizygous carriers of rare pLOF or bLOF variants (35.2 [20.3] years), potentially accounting for the lack of replication of this finding by other studies including older patients [11, 21–23]. Consistent with this result, we recently found that ~ 10% of children hospitalized for COVID-19 pneumonia carry recessive inborn errors of type I IFN immunity [19]. In addition, older patients are more likely to carry auto-Abs against type I IFN, and unlike previous studies, we excluded patients carrying such antibodies from this analysis. None of the 234 patients with critical COVID-19 excluded from this study due to the presence of auto-Abs against type I IFN carried a rare pLOF variant of the 15 genes. Hence, samples in which the vast majority of patients are over the age of 60 years and of unknown status for auto-Abs against type I IFNs would have a much lower power to identify these rare inborn errors of type I IFN immunity.

## Conclusions

Rare autosomal inborn errors of type I IFN-dependent immunity to influenza viruses can underlie critical forms of COVID-19, especially in subjects below 60 years of age, in addition to X-linked TLR7 deficiency. The search for additional rare mutations conferring a strong predisposition to life-threatening COVID-19 should focus on young patients with critical COVID-19 without auto-Abs against type I IFNs.

## Supplementary Information

**Additional file 1:** Supplementary Methods. **Fig S1.** Luciferase assay on HEK293T cells transfected with the pGL4.32 luciferase reporter construct and an expression vector for *Renilla* luciferase together with no vector (mock), EV, WT, or 4 *TLR7* variants found in our cohort. After 24 h, transfected cells were left untreated or were treated by incubation with 1 μg/mL R848 for 24 h. These data were established from two independent experiments. The *y*-axis represents NF-κB transcriptional activity as a percentage of the WT. The *x*-axis indicates the alleles used for transfection. **Fig S2.** Age distribution as boxplot and violin plot of the critical COVID-19 cases according to the carrier status of pLOF/bLOF at 15 type I IFN-related loci. Mean age of the patients for each category is shown in red. T-test was used to compare the means, showing a significant difference between non-carriers and carriers of heterozygous or homozygous/hemizygous variants (*P* = 2.2 × 10^−6^) and between heterozygous carriers and homozygous/hemizygous carriers (*P* = 0.008). **Fig S3.** Empirical power of our sample to detect an association at the 2.5 × 10^−6^ exome-wide significance threshold for various relative risks and proportion of carriers of at least one disease causing variant in the general population (PD), as estimated by simulation study (*N* = 1000 replicates).

**Additional file 2: Table S1.** Number of genes tested and Genomic inflation factor for each model and variant set. **Table S2.** Complete results of the genome-wide burden joint analysis, trans-pipeline meta-analysis and trans-ethnic meta-analysis on rare variants. **Table S3.** Best results of the genome-wide burden analysis on rare variants under a co-dominant and dominant model. **Table S4.** TLR7 homozygous and hemizygous variants (AF < 0.01). **Table S5.** Results of the genome-wide burden analysis on common and rare variants under a co-dominant model. **Table S6.** Results of the genome-wide burden analyses for the candidate genes identified by GWAS under co-dominant model. **Table S7.** Characteristics of patients and controls in the full sample and according to the inclusion in the Zhang Q. et al., Science 2020 paper. **Table S8.** Carriers of rare pLOF/bLOF variants in genes involved in type I IFN immunity to influenza virus. **Table S9.** Branchpoint variants identified by BPHunter and characteristics of the carriers. **Table S10.** Age and sex stratified analysis for the 15 type I IFN-related loci. **Table S11.** Enrichment analysis of rare variants, including missense and inframe variants, in genes involved in type I IFN immunity in the full cohort of 3269 cases and 1373 controls. **Table S12.** pLI and CoNeS distribution of the analyzed genes.

## Data Availability

Data supporting the findings of this study are available within the manuscript and supplemental files. The whole-genome sequencing data of anonymized patients recruited through the National Institutes of Health (NIH) and sequenced at the National Institute of Allergy and Infectious Diseases (NIAID) through the Uniformed Services University of the Health Sciences (USUHS)/the American Genome Center (TAGC) are available under dbGaP submission phs002245.v1. Other patients were not consented to share the raw WES/WGS data files beyond the research and clinical teams.
