# Correction: Rare predicted loss-of-function variants of type I IFN immunity genes are associated with life-threatening COVID-19

**DOI:** 10.1186/s13073-023-01278-0

**Published:** 2024-01-06

**Authors:** Daniela Matuozzo, Estelle Talouarn, Astrid Marchal, Peng Zhang, Jeremy Manry, Yoann Seeleuthner, Yu Zhang, Alexandre Bolze, Matthieu Chaldebas, Baptiste Milisavljevic, Adrian Gervais, Paul Bastard, Takaki Asano, Lucy Bizien, Federica Barzaghi, Hassan Abolhassani, Ahmad Abou Tayoun, Alessandro Aiuti, Ilad Alavi Darazam, Luis M. Allende, Rebeca Alonso-Arias, Andrés Augusto Arias, Gokhan Aytekin, Peter Bergman, Simone Bondesan, Yenan T. Bryceson, Ingrid G. Bustos, Oscar Cabrera-Marante, Sheila Carcel, Paola Carrera, Giorgio Casari, Khalil Chaïbi, Roger Colobran, Antonio Condino-Neto, Laura E. Covill, Ottavia M. Delmonte, Loubna El Zein, Carlos Flores, Peter K. Gregersen, Marta Gut, Filomeen Haerynck, Rabih Halwani, Selda Hancerli, Lennart Hammarström, Nevin Hatipoğlu, Adem Karbuz, Sevgi Keles, Christèle Kyheng, Rafael Leon-Lopez, Jose Luis Franco, Davood Mansouri, Javier Martinez-Picado, Ozge Metin Akcan, Isabelle Migeotte, Pierre-Emmanuel Morange, Guillaume Morelle, Andrea Martin-Nalda, Giuseppe Novelli, Antonio Novelli, Tayfun Ozcelik, Figen Palabiyik, Qiang Pan-Hammarström, Rebeca Pérez de Diego, Laura Planas-Serra, Daniel E. Pleguezuelo, Carolina Prando, Aurora Pujol, Luis Felipe Reyes, Jacques G. Rivière, Carlos Rodriguez-Gallego, Julian Rojas, Patrizia Rovere-Querini, Agatha Schlüter, Mohammad Shahrooei, Ali Sobh, Pere Soler-Palacin, Yacine Tandjaoui-Lambiotte, Imran Tipu, Cristina Tresoldi, Jesus Troya, Diederik van de Beek, Mayana Zatz, Pawel Zawadzki, Saleh Zaid Al-Muhsen, Mohammed Faraj Alosaimi, Fahad M. Alsohime, Hagit Baris-Feldman, Manish J. Butte, Stefan N. Constantinescu, Megan A. Cooper, Clifton L. Dalgard, Jacques Fellay, James R. Heath, Yu-Lung Lau, Richard P. Lifton, Tom Maniatis, Trine H. Mogensen, Horst von Bernuth, Alban Lermine, Michel Vidaud, Anne Boland, Jean-François Deleuze, Robert Nussbaum, Amanda Kahn-Kirby, France Mentre, Sarah Tubiana, Guy Gorochov, Florence Tubach, Pierre Hausfater, COVID Human Genetic Effort, Laurent Abel, Saleh Al-Muhsen, Fahd Al-Mulla, Mark S. Anderson, Evangelos Andreakos, Andrés A. Arias, Hagit Baris Feldman, Alexandre Belot, Catherine M. Biggs, Dusan Bogunovic, Anastasiia Bondarenko, Ahmed A. Bousfiha, Petter Brodin, Yenan Bryceson, Carlos D. Bustamante, Samya Chakravorty, John Christodoulou, Murkesh Desai, Beth A. Drolet, Jamila El Baghdadi, Sara Espinosa-Padilla, José Luis Franco, Antoine Froidure, David Hagin, Sarah E. Henrickson, Elena W. Y. Hsieh, Eystein Husebye, Kohsuke Imai, Yuval Itan, Erich D. Jarvis, Timokratis Karamitros, Kai Kisand, Cheng-Lung Ku, Yun Ling, Carrie L. Lucas, László Maródi, Isabelle Meyts, Joshua D. Milner, Kristina Mironska, Tomohiro Morio, Lisa F. P. Ng, Luigi D. Notarangelo, Cliona O’Farrelly, Satoshi Okada, Anna M. Planas, Lluis Quintana-Murci, Laurent Renia, Igor Resnick, Carlos Rodríguez-Gallego, Vanessa Sancho-Shimizu, Anna Sediva, Mikko R. J. Seppänen, Anna Shcherbina, Ondrej Slaby, Andrew L. Snow, Pere Soler-Palacín, András N. Spaan, Ivan Tancevski, Stuart G. Tangye, Sathishkumar Ramaswamy, Stuart E. Turvey, Furkan Uddin, Mohammed J. Uddin, Diederik van de Beek, Donald C. Vinh, Mayana Zatz, Helen C. Su, Jean-Laurent Casanova, COVIDeF Study Group, Serge Bureau, Yannick Vacher, Anne Gysembergh-Houal, Lauren Demerville, Abla Chachoua, Sebastien Abad, Radhiya Abassi, Abdelrafie Abdellaoui, Abdelkrim Abdelmalek, Hendy Abdoul, Helene Abergel, Fariza Abeud, Sophie Abgrall, Noemie Abisror, Marylise Adechian, Nordine Aderdour, Hakeem Farid Admane, Frederic Adnet, Sara Afritt, Helene Agostini, Claire Aguilar, Sophie Agut, Tommaso Francesco Aiello, Marc Ait Kaci, Hafid Ait Oufella, Gokula Ajeenthiravasan, Virginie Alauzy, Fanny Alby-Laurent, Lucie Allard, Marie-Alexandra Alyanakian, Blanca Amador Borrero, Sabrina Amam, Lucile Amrouche, Marc Andronikof, Dany Anglicheau, Nadia Anguel, Djillali Annane, Mohammed Aounzou, Caroline Aparicio, Gladys Aratus, Jean-Benoit Arlet, Jeremy Arzoine, Elisabeth Aslangul, Mona Assefi, Adeline Aubry, Laetitia Audiffred, Etienne Audureau, Christelle Nathalie Auger, Jean-Charles Auregan, Celine Awotar, Sonia Ayllon Milla, Delphine Azan, Laurene Azemar, Billal Azzouguen, Marwa Bachir Elrufaai, Aïda Badsi, Prissile Bakouboula, Coline Balcerowiak, Fanta Balde, Elodie Baldivia, Eliane-Flore Bangamingo, Amandine Baptiste, Fanny Baran-Marszak, Caroline Barau, Nathalie Barget, Flore Baronnet, Romain Barthelemy, Jean-Luc Baudel, Camille Baudry, Elodie Baudry, Laurent Beaugerie, Adel Belamri, Nicolas Belaube, Rhida Belilita, Pierre Bellassen, Rawan Belmokhtar, Isabel Beltran, Ruben Benainous, Mourad Benallaoua, Robert Benamouzig, Amélie Benbara, Jaouad Benhida, Anis Benkhelouf, Jihene Benlagha, Chahinez Benmostafa, Skander Benothmane, Miassa Bentifraouine, Laurence Berard, Quentin Bernier, Enora Berti, Astrid Bertier, Laure Berton, Simon Bessis, Alexandra Beurton, Celine Bianco, Clara Bianquis, Frank Bidar, Philippe Blanche, Clarisse Blayau, Alexandre Bleibtreu, Emmanuelle Blin, Coralie Bloch-Queyrat, Marie-Christophe Boissier, Diane Bollens, Marion Bolzoni, Rudy pierre Bompard, Nicolas Bonnet, Justine Bonnouvrier, Shirmonecrystal Botha, Wissam Boucenna, Fatiha Bouchama, Olivier Bouchaud, Hanane Bouchghoul, Taoueslylia Boudjebla, Noel Boudjema, Catherine Bouffard, Adrien Bougle, Meriem Bouguerra, Leila Bouras, Agnes Bourcier, Anne Bourgarit Durand, Anne Bourrier, Fabrice Bouscarat, Diane Bouvry, Nesrine Bouziri, Ons Bouzrara, Sarah Bribier, Delphine Brugier, Melanie Brunel, Eida Bui, Anne Buisson, Iryna Bukreyeva, Côme Bureau, Jacques Cadranel, Johann Cailhol, Ruxandra Calin, Clara Campos Vega, Pauline Canavaggio, Marta Cancella, Delphine Cantin, Albert Cao, Lionel Carbillon, Nicolas Carlier, Clementine Cassard, Guylaine Castor, Marion Cauchy, Olivier Cha, Benjamin Chaigne, Salima Challal, Karine Champion, Patrick Chariot, Julie Chas, Simon Chauveau, Anthony Chauvin, Clement Chauvin, Nathalie Chavarot, Kamélia Chebbout, Mustapha Cherai, Ilaria Cherubini, Amelie Chevalier, Thibault Chiarabini, Thierry Chinet, Richard Chocron, Pascaline Choinier, Juliette Chommeloux, Christophe Choquet, Laure Choupeaux, Benjamin Chousterman, Dragosmarius Ciocan, Ada Clarke, Gaëlle Clavere, Florian Clavier, Karine Clement, Sebastien Clerc, Yves Cohen, Fleur Cohen, Adrien Cohen, Audrey Coilly, Hester Colboc, Pauline Colin, Magalie Collet, Chloé Comarmond, Emeline Combacon, Alain Combes, Celine Comparon, Jean-Michel Constantin, Hugues Cordel, Anne-Gael Cordier, Adrien Costantini, Nathalie Costedoat Chalumeau, Camille Couffignal, Doriane Coupeau, Alain Creange, Yannie Cuvillier Lamarre, Charlène Da Silveira, Sandrine Dautheville Guibal El Kayani, Nathalie De Castro, Yann De Rycke, Lucie Del Pozo, Quentin Delannoy, Mathieu Delay, Robin Deleris, Juliette Delforge, Laëtitia Delphine, Noemie Demare, Sophie Demeret, Alexandre Demoule, Aurore Deniau, François Depret, Sophie Derolez, Ouda Derradji, Nawal Derridj, Vincent Descamps, Lydia Deschamps, Celine Desconclois, Cyrielle Desnos, Karine Desongins, Robin Dhote, Benjamin Diallo, Morgane Didier, Myriam Diemer, Stephane Diez, Juliette Djadi-Prat, Fatima-Zohra Djamouri Monnory, Siham Djebara, Naoual Djebra, Minette Djietcheu, Hadjer Djillali, Nouara Djouadi, Severine Donneger, Catarina Dos Santos, Nathalie Dournon, Martin Dres, Laura Droctove, Marie Drogrey, Margot Dropy, Elodie Drouet, Valérie Dubosq, Evelyne Dubreucq, Estelle Dubus, Boris Duchemann, Thibault Duchenoy, Emmanuel Dudoignon, Romain Dufau, Florence Dumas, Clara Duran, Emmanuelle Duron, Antoine Durrbach, Claudine Duvivier, Nathan Ebstein, Jihane El Khalifa, Alexandre Elabbadi, Caroline Elie, Gabriel Ernotte, Anne Esling, Martin Etienne, Xavier Eyer, Muriel sarah Fartoukh, Takoua Fayali, Marion Fermaut, Arianna Fiorentino, Souha Fliss, Marie-Céline Fournier, Benjamin Fournier, Hélène Francois, Olivia Freynet, Yvann Frigout, Isaure Fromont, Axelle Fuentes, Thomas Furet, Joris Galand, Marc Garnier, Agnes Gaubert, Stéphane Gaudry, Samuel Gaugain, Damien Gauthier, Maxime Gautier, Sophie Georgin-Lavialle, Daniela Geromin, Mohamed Ghalayini, Bijan Ghaleh, Myriam Ghezal, Aude Gibelin, Linda Gimeno, Benoit Girard, Bénédicte Giroux Leprieur, Doryan Gomes, Elisabete Gomes-Pires, Anne Gouge, Amel Gouja, Helene Goulet, Sylvain Goupil, Jeanne Goupil De Bouille, Julien Gras, Segolene Greffe, Lamiae Grimaldi, Paul Guedeney, Bertrand Guidet, Matthias Guillo, Mariechristelle Gulczynski, Tassadit Hadjam, Didier Haguenauer, Soumeya Hammal, Nadjib Hammoudi, Olivier Hanon, Anarole Harrois, Coraline Hautem, Guillaume Hekimian, Nicholas Heming, Olivier Hermine, Sylvie Ho, Marie Houllier, Benjamin Huot, Tessa Huscenot, Wafa Ibn Saied, Ghilas Ikherbane, Meriem Imarazene, Patrick Ingiliz, Lina Iratni, Stephane Jaureguiberry, Jean-Francois Jean-Marc, Deleena Jeyarajasingham, Pauline Jouany, Veronique Jouis, Clement Jourdaine, Ouifiya Kafif, Rim Kallala, Sandrine Katsahian, Lilit Kelesyan, Vixra Keo, Flora Ketz, Warda Khamis, Enfel Khelili, Mehdi Khellaf, Christy Gaëlla Kotokpo Youkou, Ilias Kounis, Gaelle Kpalma, Jessica Krause, Vincent Labbe, Karine Lacombe, Jean-Marc Lacorte, Anne Gaelle Lafont, Emmanuel Lafont, Lynda Lagha, Lionel Lamhaut, Aymeric Lancelot, Cecilia Landman, Fanny Lanternier, Cecile Larcheveque, Caroline Lascoux Combe, Ludovic Lassel, Benjamin Laverdant, Christophe Lavergne, Jean-Rémi Lavillegrand, Pompilia Lazureanu, Loïc Le Guennec, Lamia Leberre, Claire Leblanc, Marion Leboyer, Francois Lecomte, Marine Lecorre, Romain Leenhardt, Marylou Lefebvre, Bénédicte Lefebvre, Paul Legendre, Anne Leger, Laurence Legros, Justyna Legrosse, Sébastien Lehuunghia, Julien Lemarec, Jeremie Leporrier-Ext, Manon Lesein, Hubert Lesur, Vincent Levy, Albert Levy, Edwige Lopes, Amanda Lopes, Vanessa Lopez, Julien Lopinto, Olivier Lortholary, Badr Louadah, Bénédicte Loze, Marie-Laure Lucas, Axelle Lucasamichi, Liem Binh Luong, Arouna Magazimama-Ext, David Maingret, Lakhdar Mameri, Philippe Manivet, Cylia Mansouri, Estelle Marcault, Jonathan Marey, Nathalie Marin, Clémence Marois, Olivier Martin, Lou Martineau, Cannelle Martinez-Lopez, Pierre Martyniuck, Pauline Mary De Farcy, Nessrine Marzouk, Rafik Masmoudi, Alexandre Mebazaa, Frédéric Mechai, Fabio Mecozzi, Chamseddine Mediouni, Bruno Megarbane, Mohamed Meghadecha, Élodie Mejean, Arsene Mekinian, Nour Mekki Abdelhadi, Rania Mekni, Thinhinan Sabrina Meliti, Breno Melo Lima, Paris Meng, Soraya Merbah, Fadhila Messani, Yasmine Messaoudi, Baboo-Irwinsingh Mewasing, Lydia Meziane, Carole Michelot-Burger, Françoise Mignot, Fadi Hillary Minka, Makoto Miyara, Pierre Moine, Jean-Michel Molina, Anaïs Montegnies-Boulet, Alexandra Monti, Claire Montlahuc, Anne-Lise Montout, Alexandre Moores, Caroline Morbieu, Helene Mortelette, Stéphane Mouly, Rosita Muzaffar, Cherifa Iness Nacerddine, Marine Nadal, Hajer Nadif, Kladoum Nassarmadji, Pierre Natella, Sandrine Ndingamondze, Stefan Neraal, Caroline Nguyen, Bao N’Guyen, Isabelle Nion Larmurier, Luc Nlomenyengue, Nicolas Noel, Hilario Nunes, Edris Omar, Zineb Ouazene, Elise Ouedraogo, Wassila Ouelaa, Anissa Oukhedouma, Yasmina Ould Amara, Herve Oya, Johanna Oziel, Thomas Padilla, Elena Paillaud, Solenne Paiva, Beatrice Parfait, Perrine Parize, Christophe Parizot, Antoine Parrot, Arthur Pavot, Laetitia Peaudecerf, Frédéric Pene, Marion Pepin, Julie Pernet, Claire Pernin, Mylène Petit, Olivier Peyrony, Marie-Pierre Pietri, Olivia Pietri, Marc Pineton De Chambrun, Michelle Pinson, Claire Pintado, Valentine Piquard, Christine Pires, Benjamin Planquette, Sandrine Poirier, Anne-Laure Pomel, Stéphanie Pons, Diane Ponscarme, Annegaelle Pourcelot, Valérie Pourcher, Anne Pouvaret, Florian Prever, Miresta Previlon, Margot Prevost, Marie-Julie Provoost, Cyril Quemeneur, Cédric Rafat, Agathe Rami, Brigitte Ranque, Maurice Raphael, Jean Herle Raphalen, Anna Rastoin, Mathieu Raux, Amani Rebai, Michael Reby, Alexis Regent, Asma Regrag, Matthieu Resche-Rigon, Quentin Ressaire, Christian Richard, Mariecaroline Richard, Maxence Robert, Benjamin Rohaut, Camille Rolland-Debord, Jacques Ropers, Anne-Marie Roque-Afonso, Charlotte Rosso, Mélanie Rousseaux, Nabila Rousseaux, Swasti Roux, Lorène Roux, Claire Rouzaud, Antoine Rozes, Emma Rubenstein, Jean-Marc Sabate, Sheila Sabet, Sophie-Caroline Sacleux, Nathalie Saidenberg Kermanach, Faouzi Saliba, Dominique Salmon, Laurent Savale, Guillaume Savary, Rebecca Sberro, Anne Scemla, Frederic Schlemmer, Mathieu Schwartz, Saïd Sedfi, Samia Sefir-Kribel, Philippe Seksik, Pierre Sellier, Agathe Selves, Nicole Sembach, Luca Semerano, Marie-Victoire Senat, Damien Sene, Alexandra Serris, Lucile Sese, Naima Sghiouar, Johanna Sigaux, Martin Siguier, Johanne Silvain, Noémie Simon, Tabassome Simon, Lina Innes Skandri, Miassa Slimani, Aurélie Snauwaert, Harry Sokol, Heithem Soliman, Nisrine Soltani, Benjamin Soyer, Gabriel Steg, Lydia Suarez, Tali-Anne Szwebel, Kossi Taffame, Claire Tantet, Mariagrazia Tateo, Igor Theodose, Pierre clement Thiebaud, Caroline Thomas, Kelly Tiercelet, Julie Tisserand, Carole Tomczak, Krystel Torelino, Fatima Touam-Ext, Lilia Toumi, Gustave Toury, Mireille Toy-Miou, Olivia Tran Dinh Thanh Lien, Alexy Trandinh, Jean-Marc Treluyer, Baptiste Trinque, Jennifer Truchot, Simone Tunesi, Matthieu Turpin, Agathe Turpin, Tomas Urbina, Rafael Usubillaga Narvaez, Yurdagul Uzunhan, Prabakar Vaittinadaayar, Arnaud Valent, Maelle Valentian, Nadia Valin, Hélène Vallet, Marina Vaz, Miguel-Alejandro Vazquezibarra, Benoit Vedie, Laetitia Velly, Celine Verstuyft, Cedric Viallette, Eric Vicaut, Dorothee Vignes, Damien Vimpere, Myriam Virlouvet, Guillaume Voiriot, Lena Voisot, Emmanuel Weiss, Nicolas Weiss, Anaïs Winchenne, Youri Yordanov, Lara Zafrani, Mohamad Zaidan, Wissem Zaidi, Cathia Zak, Aida Zarhrate-Ghoul, Ouassila Zatout, Suzanne Zeino, Michel Zeitouni, Naïma Zemirli, Lorene Zerah, Ounsa Zia, Marianne Ziol, Oceane Zolario, Julien Zuber, French COVID Cohort Study Group, Laurent Abel, Claire Andrejak, François Angoulvant, Delphine Bachelet, Marie Bartoli, Romain Basmaci, Sylvie Behilill, Marine Beluze, Dehbia Benkerrou, Krishna Bhavsar, Lila Bouadma, Sabelline Bouchez, Maude Bouscambert, Minerva Cervantes-Gonzalez, Anissa Chair, Catherine Chirouze, Alexandra Coelho, Camille Couffignal, Sandrine Couffin-Cadiergues, Eric d’Ortenzio, Marie-Pierre Debray, Lauren Deconinck, Dominique Deplanque, Diane Descamps, Mathilde Desvallée, Alpha Diallo, Alphonsine Diouf, Céline Dorival, François Dubos, Xavier Duval, Brigitte Elharrar, Philippine Eloy, Vincent Enouf, Hélène Esperou, Marina Esposito-Farese, Manuel Etienne, Eglantine Ferrand Devouge, Nathalie Gault, Alexandre Gaymard, Jade Ghosn, Tristan Gigante, Morgane Gilg, Jérémie Guedj, Alexandre Hoctin, Isabelle Hoffmann, Ikram Houas, Jean-Sébastien Hulot, Salma Jaafoura, Ouifiya Kafif, Florentia Kaguelidou, Sabrina Kali, Antoine Khalil, Coralie Khan, Cédric Laouénan, Samira Laribi, Minh Le, Quentin Le Hingrat, Soizic Le Mestre, Hervé Le Nagard, François-Xavier Lescure, Sophie Letrou, Yves Levy, Bruno Lina, Guillaume Lingas, Jean Christophe Lucet, Denis Malvy, Marina Mambert, France Mentré, Amina Meziane, Hugo Mouquet, Jimmy Mullaert, Nadège Neant, Duc Nguyen, Marion Noret, Saad Nseir, Aurélie Papadopoulos, Christelle Paul, Nathan Peiffer-Smadja, Thomas Perpoint, Ventzislava Petrov-Sanchez, Gilles Peytavin, Huong Pham, Olivier Picone, Valentine Piquard, Oriane Puéchal, Christian Rabaud, Manuel Rosa-Calatrava, Bénédicte Rossignol, Patrick Rossignol, Carine Roy, Marion Schneider, Richa Su, Coralie Tardivon, Marie-Capucine Tellier, François Téoulé, Olivier Terrier, Jean-François Timsit, Christelle Tual, Sylvie Van Der Werf, Noémie Vanel, Aurélie Veislinger, Benoit Visseaux, Aurélie Wiedemann, Yazdan Yazdanpanah, CoV-Contact Cohort, Loubna Alavoine, Sylvie Behillil, Charles Burdet, Charlotte Charpentier, Aline Dechanet, Diane Descamps, Xavier Duval, Jean-Luc Ecobichon, Vincent Enouf, Wahiba Frezouls, Nadhira Houhou, Ouifiya Kafif, Jonathan Lehacaut, Sophie Letrou, Bruno Lina, Jean-Christophe Lucet, Pauline Manchon, Mariama Nouroudine, Valentine Piquard, Caroline Quintin, Michael Thy, Sylvie van der Werf, Valérie Vignali, Benoit Visseaux, Yazdan Yazdanpanah, Abir Chahine, Nawal Waucquier, Maria-Claire Migaud, Dominique Deplanque, Félix Djossou, Mayka Mergeay-Fabre, Aude Lucarelli, Magalie Demar, Léa Bruneau, Patrick Gérardin, Adrien Maillot, Christine Payet, Bruno Laviolle, Fabrice Laine, Christophe Paris, Mireille Desille-Dugast, Julie Fouchard, Denis Malvy, Duc Nguyen, Thierry Pistone, Pauline Perreau, Valérie Gissot, Carole L. E. Goas, Samatha Montagne, Lucie Richard, Catherine Chirouze, Kévin Bouiller, Maxime Desmarets, Alexandre Meunier, Marilou Bourgeon, Benjamin Lefévre, Hélène Jeulin, Karine Legrand, Sandra Lomazzi, Bernard Tardy, Amandine Gagneux-Brunon, Frédérique Bertholon, Elisabeth Botelho-Nevers, Kouakam Christelle, Leturque Nicolas, Layidé Roufai, Karine Amat, Sandrine Couffin-Cadiergues, Hélène Espérou, Samia Hendou, Giuseppe Foti, Giacomo Bellani, Giuseppe Citerio, Ernesto Contro, Alberto Pesci, Maria Grazia Valsecchi, Marina Cazzaniga, COVID Clinicians, Jorge Abad, Giulia Accordino, Cristian Achille, Sergio Aguilera-Albesa, Aina Aguiló-Cucurull, Esra Akyüz Özkan, Jonathan Antonio Roblero Albisures, Juan C. Aldave, Miquel Alfonso Ramos, Taj Ali Khan, Anna Aliberti, Seyed Alireza Nadji, Gulsum Alkan, Suzan A. AlKhater, Jerome Allardet-Servent, Mohammed S. Alshahrani, Laia Alsina, Marie-Alexandra Alyanakian, Blanca Amador Borrero, Zahir Amoura, Arnau Antolí, Romain Arrestier, Mélodie Aubart, Teresa Auguet, Iryna Avramenko, Gökhan Aytekin, Axelle Azot, Seiamak Bahram, Fanny Bajolle, Fausto Baldanti, Aurélie Baldolli, Maite Ballester, Hagit Baris Feldman, Benoit Barrou, Sabrina Basso, Gulsum Iclal Bayhan, Alexandre Belot, Liliana Bezrodnik, Agurtzane Bilbao, Geraldine Blanchard-Rohner, Ignacio Blanco, Adeline Blandinières, Daniel Blázquez-Gamero, Alexandre Bleibtreu, Marketa Bloomfield, Mireia Bolivar-Prados, Anastasiia Bondarenko, Alessandro Borghesi, Raphael Borie, Elisabeth Botdhlo-Nevers, Ahmed A. Bousfiha, Aurore Bousquet, David Boutolleau, Claire Bouvattier, Oksana Boyarchuk, Juliette Bravais, M. Luisa Briones, Marie-Eve Brunner, Raffaele Bruno, Maria Rita P. Bueno, Huda Bukhari, Jacinta Bustamante, Juan José Cáceres Agra, Ruggero Capra, Raphael Carapito, Maria Carrabba, Carlos Casasnovas, Marion Caseris, Irene Cassaniti, Martin Castelle, Francesco Castelli, Martín Castillo de Vera, Mateus V. Castro, Emilie Catherinot, Jale Bengi Celik, Alessandro Ceschi, Martin Chalumeau, Bruno Charbit, Matthew P. Cheng, Père Clavé, Bonaventura Clotet, Anna Codina, Yves Cohen, Cloé Comarmond, Alain Combes, Patrizia Comoli, Angelo G. Corsico, Betul Sozeri, Taner Coşkuner, Aleksandar Cvetkovski, Cyril Cyrus, David Dalmau, François Danion, David Ross Darley, Vincent Das, Nicolas Dauby, Stéphane Dauger, Paul De Munter, Loic de Pontual, Amin Dehban, Geoffroy Delplancq, Alexandre Demoule, Isabelle Desguerre, Antonio Di Sabatino, Jean-Luc Diehl, Stephanie Dobbelaere, Elena Domínguez-Garrido, Clément Dubost, Olov Ekwall, Şefika Elmas Bozdemir, Marwa H. Elnagdy, Melike Emiroglu, Akifumi Endo, Emine Hafize Erdeniz, Selma Erol Aytekin, Maria Pilar Etxart Lasa, Romain Euvrard, Giovanna Fabio, Laurence Faivre, Antonin Falck, Muriel Fartoukh, Morgane Faure, Miguel Fernandez Arquero, Ricard Ferrer, Jose Ferreres, Bruno Francois, Victoria Fumadó, Kitty S. C. Fung, Francesca Fusco, Alenka Gagro, Blanca Garcia Solis, Pierre Garçon, Pascale Gaussem, Zeynep Gayretli, Juana Gil-Herrera, Laurent Gilardin, Audrey Giraud Gatineau, Mònica Girona-Alarcón, Karen Alejandra Cifuentes Godínez, Jean-Christophe Goffard, Nacho Gonzales, Luis I. Gonzalez-Granado, Rafaela González-Montelongo, Antoine Guerder, Belgin Gülhan, Victor Daniel Gumucio, Leif Gunnar Hanitsch, Jan Gunst, Jérôme Hadjadj, Tetyana Hariyan, Nevin Hatipoglu, Deniz Heppekcan, Elisa Hernandez-Brito, Po-ki Ho, María Soledad Holanda-Peña, Juan P. Horcajada, Sami Hraiech, Linda Humbert, Ivan F. N. Hung, Alejandro D. Iglesias, Antonio Íñigo-Campos, Matthieu Jamme, María Jesús Arranz, Marie-Thérèse Jimeno, Iolanda Jordan, Saliha Kanik-Yüksek, Yalcin Burak Kara, Aydın Karahan, Kadriye Kart Yasar, Ozgur Kasapcopur, Kenichi Kashimada, Yasemin Kendir Demirkol, Yasutoshi Kido, Can Kizil, Ahmet Osman Kılıç, Adam Klocperk, Antonia Koutsoukou, Zbigniew J. Król, Hatem Ksouri, Paul Kuentz, Arthur M. C. Kwan, Yat Wah M. Kwan, Janette S. Y. Kwok, Jean-Christophe Lagier, David S. Y. Lam, Vicky Lampropoulou, Fanny Lanternier, Fleur Le Bourgeois, Yee-Sin Leo, Rafael Leon Lopez, Daniel Leung, Michael Levin, Michael Levy, Romain Lévy, Zhi Li, Daniele Lilleri, Edson Jose Adrian Bolanos Lima, Agnes Linglart, Eduardo López-Collazo, José M. Lorenzo-Salazar, Céline Louapre, Catherine Lubetzki, Kwok-Cheung Lung, Charles-Edouard Luyt, David C. Lye, Cinthia Magnone, Enrico Marchioni, Carola Marioli, Majid Marjani, Laura Marques, Jesus Marquez Pereira, Andrea Martín-Nalda, David Martínez Pueyo, Iciar Marzana, Carmen Mata-Martínez, Alexis Mathian, Larissa R. B. Matos, Gail V. Matthews, Julien Mayaux, Raquel McLaughlin-Garcia, Philippe Meersseman, Jean-Louis Mège, Armand Mekontso-Dessap, Isabelle Melki, Federica Meloni, Jean-François Meritet, Paolo Merlani, Özge Metin Akcan, Isabelle Meyts, Mehdi Mezidi, Maude Millereux, Matthieu Million, Tristan Mirault, Clotilde Mircher, Mehdi Mirsaeidi, Yoko Mizoguchi, Bhavi P. Modi, Francesco Mojoli, Elsa Moncomble, Abián Montesdeoca Melián, Antonio Morales Martinez, Francisco Morandeira, Cléemence Mordacq, Stéphane J. Mouly, Adrián Muñoz-Barrera, Cyril Nafati, Shintaro Nagashima, Yu Nakagama, Bénédicte Neven, João Farela Neves, Lisa F. P. Ng, Yuk-Yung Ng, hubert Nielly, Yeray Novoa Medina, Esmeralda Nuñez Cuadros, J. Gonzalo Ocejo-Vinyals, Keisuke Okamoto, Mehdi Oualha, Amani Ouedrani, Tayfun Özçelik, Aslinur Ozkaya-Parlakay, Michele Pagani, Maria Papadaki, Christophe Parizot, Philippe Parola, Tiffany Pascreau, Stéphane Paul, Estela Paz-Artal, Sigifredo Pedraza, Nancy Carolina González Pellecer, Silvia Pellegrini, Xosé Luis Pérez-Fernández, Aurélien Philippe, Quentin Philippot, Adrien Picod, Marc Pineton de Chambrun, Antonio Piralla, Dominique Ploin, Julien Poissy, Géraldine Poncelet, Garyphallia Poulakou, Marie S. Pouletty, Persia Pourshahnazari, Jia Li Qiu-Chen, Paul Quentric, Thomas Rambaud, Didier Raoult, Violette Raoult, Anne-Sophie Rebillat, Claire Redin, Léa Resmini, Pilar Ricart, Jean-Christophe Richard, Raúl Rigo-Bonnin, Nadia rivet, Gemma Rocamora-Blanch, Mathieu P. Rodero, Carlos Rodrigo, Luis Antonio Rodriguez, Agustí Rodriguez-Palmero, Carolina Soledad Romero, Anya Rothenbuhler, Damien Roux, Nikoletta Rovina, Flore Rozenberg, Yvon Ruch, Montse Ruiz, Maria Yolanda Ruiz del Prado, Juan Carlos Ruiz-Rodriguez, Joan Sabater-Riera, Kai Saks, Maria Salagianni, Oliver Sanchez, Adrián Sánchez-Montalvá, Silvia Sánchez-Ramón, Laire Schidlowski, Agatha Schluter, Julien Schmidt, Matthieu Schmidt, Catharina Schuetz, Cyril E. Schweitzer, Francesco Scolari, Anna Sediva, Luis Seijo, Analia Gisela Seminario, Damien Sene, Piseth Seng, Sevtap Senoglu, Mikko Seppänen, Alex Serra Llovich, Anna Shcherbina, Virginie Siguret, Eleni Siouti, David M. Smadja, Nikaia Smith, Xavier Solanich, Jordi Solé-Violán, Catherine Soler, Pere Soler-Palacín, Betül Sözeri, Giulia Maria Stella, Yuriy Stepanovskiy, Annabelle Stoclin, Fabio Taccone, Jean-Luc Taupin, Simon J. Tavernier, Loreto Vidaur Tello, Benjamin Terrier, Guillaume Thiery, Christian Thorball, Karolina Thorn, Caroline Thumerelle, Martin Tolstrup, Gabriele Tomasoni, Julie Toubiana, Josep Trenado Alvarez, Vasiliki Triantafyllia, Sophie Trouillet-Assant, Jesús Troya, Owen T. Y. Tsang, Liina Tserel, Eugene Y. K. Tso, Alessandra Tucci, Şadiye Kübra Tüter Öz, Matilde Valeria Ursini, Takanori Utsumi, Yurdagul Uzunhan, Pierre Vabres, Juan Valencia-Ramos, Ana Maria Van Den Rym, Isabelle Vandernoot, Valentina Velez-Santamaria, Silvia Patricia Zuniga Veliz, Mateus C. Vidigal, Sébastien Viel, Cédric Villain, Marie E. Vilaire-Meunier, Judit Villar-García, Audrey Vincent, Guillaume Voiriot, Alla Volokha, Fanny Vuotto, Els Wauters, Joost Wauters, Alan K. L. Wu, Tak-Chiu Wu, Aysun Yahşi, Osman Yesilbas, Mehmet Yildiz, Barnaby E. Young, Ufuk Yükselmiş, Mayana Zatz, Marco Zecca, Valentina Zuccaro, Jens Van Praet, Bart N. Lambrecht, Eva Van Braeckel, Cédric Bosteels, Levi Hoste, Eric Hoste, Fré Bauters, Jozefien De Clercq, Catherine Heijmans, Hans Slabbynck, Leslie Naesens, Benoit Florkin, Cécile Boulanger, Dimitri Vanderlinden, Orchestra Working Group, Laurent Abel, Matilda Berkell, Valerio Carelli, Alessia Fiorentino, Surbi Malhotra, Alessandro Mattiaccio, Tommaso Pippucci, Marco Seri, Evelina Tacconelli, Amsterdam UMC Covid-19 Biobank, Michiel van Agtmael, Anne Geke Algera, Brent Appelman, Frank van Baarle, Diane Bax, Martijn Beudel, Harm Jan Bogaard, Marije Bomers, Peter Bonta, Lieuwe Bos, Michela Botta, Justin de Brabander, Godelieve de Bree, Sanne de Bruin, David T. P. Buis, Marianna Bugiani, Esther Bulle, Osoul Chouchane Alex Cloherty, Mirjam Dijkstra, Dave A. Dongelmans, Romein W. G. Dujardin, Paul Elbers, Lucas Fleuren, Suzanne Geerlings Theo Geijtenbeek, Armand Girbes, Bram Goorhuis, Martin P. Grobusch, Florianne Hafkamp, Laura Hagens, Jorg Hamann, Vanessa Harris, Robert Hemke, Sabine M. Hermans Leo Heunks, Markus Hollmann, Janneke Horn, Joppe W. Hovius, Menno D. de Jong, Rutger Koning, Endry H. T. Lim, Niels van Mourik, Jeaninne Nellen, Esther J. Nossent, Frederique Paulus, Edgar Peters, Dan A. I. Pina-Fuentes, Tom van der Poll, Bennedikt Preckel, Jan M. Prins, Jorinde Raasveld, Tom Reijnders, Maurits C. F. J. de Rotte, Michiel Schinkel, Marcus J. Schultz, Femke A. P. Schrauwen, Alex Schuurmans, Jaap Schuurmans, Kim Sigaloff, Marleen A. Slim, Patrick Smeele, Marry Smit, Cornelis S. Stijnis, Willemke Stilma, Charlotte Teunissen, Patrick Thoral, Anissa M. Tsonas, Pieter R. Tuinman, Marc van der Valk, Denise Veelo, Carolien Volleman, Heder de Vries, Lonneke A. Vught, Michèle van Vugt, Dorien Wouters, A. H. Zwinderman, Matthijs C. Brouwer, W. Joost Wiersinga, Alexander P. J. Vlaar, Diederik van de Beek, NIAID-USUHS COVID Study Group, Miranda F. Tompkins, Camille Alba, Andrew L. Snow, Daniel N. Hupalo, John Rosenberger, Gauthaman Sukumar, Matthew D. Wilkerson, Xijun Zhang, Justin Lack, Andrew J. Oler, Kerry Dobbs, Jeffrey J. Danielson, Andrea Biondi, Laura Rachele Bettini, Mariella D’Angio’, Ilaria Beretta, Luisa Imberti, Alessandra Sottini, Virginia Quaresima, Eugenia Quiros-Roldan, Camillo Rossi, Isabelle Meyts, Shen-Ying Zhang, Anne Puel, Luigi D. Notarangelo, Stephanie Boisson-Dupuis, Helen C. Su, Bertrand Boisson, Emmanuelle Jouanguy, Jean-Laurent Casanova, Qian Zhang, Laurent Abel, Aurélie Cobat

**Affiliations:** 1 Laboratory of Human Genetics of Infectious Diseases, Necker Branch, INSERM U1163, Paris, France; 2 University Paris Cité, Imagine Institute, Paris, France; 3 St. Giles Laboratory of Human Genetics of Infectious Diseases, Rockefeller Branch, The Rockefeller University, New York, NY USA; 4 Laboratory of Clinical Immunology and Microbiology, Division of Intramural Research, NIAID, Bethesda, MD USA; 5 Helix, San Mateo, CA USA; 6 Department of Paediatric Immunohematology, IRCCS San Raffaele Scientific Institute, Milan, Italy; 7 Department of Biosciences and Nutrition, Karolinska Institute, Stockholm, Sweden; 8 Research Center for Immunodeficiencies, Pediatrics Center of Excellence, Children’s Medical Center, Tehran, Iran; 9 Al Jalila Genomics Center of Excellence, Al Jalila Children’s Specialty Hospital, Dubai, United Arab Emirates; 10 Center for Genomic Discovery, Mohammed Bin Rashid University of Medicine and Health Sciences, Dubai, United Arab Emirates; 11 San Raffaele Telethon Institute for Gene Therapy (SR-Tiget), IRCSS San Raffaele Scientific Institute, Milan, Italy; 12 Vita-Salute San Raffaele University, Milan, Italy; 13 Infectious Diseases and Tropical Medicine Research Center, Shahid Beheshti University of Medical Sciences, Tehran, Iran; 14 Department of Infectious Diseases and Tropical Medicine, Loghman Hakim Hospital, Shahid Beheshti University of Medical Sciences, Tehran, Iran; 15 Immunology Department, University Hospital 12 de Octubre, Research Institute imas12 and Complutense University, Madrid, Spain; 16 Immunology Department, Hospital Universitario Central de Asturias, Oviedo, Spain; 17 Department of Microbiology and Parasitology, Primary Immunodeficiencies Group, School of Medicine, University of Antioquia UdeA, 050010 Medellin, Colombia; 18 School of Microbiology, University of Antioquia UdeA, 050010 Medellin, Colombia; 19 Deparment of Internal Medicine, Division of Allergy and Immunology, Konya City Hospital, Konya, Turkey; 20 Department of Infectious Diseases, The Immunodeficiency Unit, Karolinska University Hospital, Stockholm, Sweden; 21 Department of Laboratory Medicine, Division of Clinical Immunology, Stockholm, Sweden; 22 Clinical Genomics, IRCSS San Raffaele Scientific Institute, Milan, Italy; 23 Department of Medicine, Centre for Hematology and Regenerative Medicine, Karolinska Institute, Stockholm, Sweden; 24 Universidad de La Sabana, Chía, Colombia; 25 Institute of Biomedical Research of IdiPAZ, University Hospital “La Paz”, Madrid, Spain; 26 Unidad de Gestión Clínica de Cuidados Intensivos, Instituto Maimónides de Investigación Biomédica de Córdoba (IMIBIC), Hospital Universitario Reina Sofía, Universidad de Córdoba (UCO), Córdoba, Spain; 27 Division of Genetics and Cell Biology, Genome-Phenome Relationship, San Raffaele Hospital, Milan, Italy; 28 School of Medicine, Vita-Salute San Raffaele University, Milan, Italy; 29 Anesthesiology and Critical Care Medicine Department, Avicenne Hospital, Assistance Publique-Hôpitaux de Paris, Bobigny, France; 30 Common and Rare Kidney Diseases, Sorbonne University, INSERM UMR-S 1155, Paris, France; 31 Jeffrey Modell Diagnostic and Research Center for Primary Immunodeficiencies, Barcelona, Catalonia Spain; 32 Translational Immunology Research Group, Vall d’Hebron Research Institute (VHIR), Vall d’Hebron University Hospital (HUVH), Vall d’Hebron Barcelona Hospital Campus, Barcelona, Catalonia Spain; 33 Genetics Department, Immunology Division, Vall d’Hebron University Hospital (HUVH), Vall d’Hebron Barcelona Hospital Campus, Autonomous University of Barcelona (UAB), Barcelona, Catalonia Spain; 34 Department of Immunology, Institute of Biomedical Sciences, University of São Paulo, São Paulo, Brazil; 35 Biology Department, Lebanese University, Beirut, Lebanon; 36 Genomics Division, Institute of Technology and Renewable Energies (ITER), Santa Cruz de Tenerife, Spain; 37 CIBER de Enfermedades Respiratorias, Carlos III Health Institute, Madrid, Spain; 38 Research Unit, University Hospital of Ntra. Sra. de Candelaria, Santa Cruz de Tenerife, Spain; 39 Faculty of Health Sciences, University of Fernando Pessoa Canarias, Las Palmas de Gran Canaria, Spain; 40 Feinstein Institute for Medical Research, Northwell Health USA, Manhasset, NY USA; 41 CNAG-CRG, Centre for Genomic Regulation (CRG), The Barcelona Institute of Science and Technology (BIST), Barcelona, Spain; 42 Department of Internal Diseases and Pediatrics, Primary Immune Deficiency Research Laboratory, Centre for Primary Immunodeficiency Ghent, Jeffrey Modell Diagnosis and Research Centre, Ghent, Belgium; 43 Sharjah Institute for Medical Research, College of Medicine, University of Sharjah, Sharjah, United Arab Emirates; 44 Department of Pediatrics (Infectious Diseases), Istanbul Faculty of Medicine, Istanbul University, Istanbul, Turkey; 45 Pediatric Infectious Diseases Unit, Bakirkoy Dr Sadi Konuk Training and Research Hospital, University of Health Sciences, Istanbul, Turkey; 46 Department of Pediatric Infectious Disease, Dr. Cemil Tascioglu City Hospital, Istanbul, Turkey; 47 Meram Medical Faculty, Division of Pediatric Allergy and Immunology, Necmettin Erbakan University, Konya, Turkey; 48 Department of General Paediatrics, Hôpital Bicêtre, Assistance Publique-Hôpitaux de Paris, University of Paris Saclay, Le Kremlin-Bicêtre, France; 49 Primary Immunodeficiencies Group, School of Microbiology, University of Antioquia UdeA, Medellin, Colombia; 50 The Clinical Tuberculosis and Epidemiology Research Center, National Research Institute of Tuberculosis and Lung Diseases, Shahid Beheshti University of Medical Sciences, Tehran, Iran; 51 Department of Clinical Immunology and Infectious Diseases, National Research Institute of Tuberculosis and Lung Diseases, Shahid Beheshti University of Medical Sciences, Tehran, Iran; 52 Pediatric Respiratory Diseases Research Center, National Research Institute of Tuberculosis and Lung Diseases, Shahid Beheshti University of Medical Sciences, Tehran, Iran; 53 IrsiCaixa AIDS Research Institute and Institute for Health Science Research Germans Trias I Pujol (IGTP), Badalona, Spain; 54 Institute for Health Science Research Germans Trias I Pujol (IGTP), Badalona, Spain; 55 Department of Infectious Diseases and Immunity, University of Vic-Central University of Catalonia, Vic, Spain; 56 Catalan Institution of Research and Advanced Studies (ICREA), Barcelona, Spain; 57 Consorcio Centro de Investigación Biomédica en Red de Enfermedades Infecciosas (CIBERINFEC), Instituto de Salud Carlos III, Madrid, Spain; 58 Centre de Génétique Humaine de L’Université Libre de Bruxelles, Hôpital Erasme, Brussels, Belgium; 59 Laboratory of Haematology, La Timone Hospital, Marseille, France; 60 C2VN, INSERM, INRAE, Aix-Marseille University, Marseille, France; 61 Pediatric Infectious Diseases and Immunodeficiencies Unit, Vall d’Hebron Research Institute (VHIR), Vall d’Hebron University Hospital (HUVH), Vall d’Hebron Barcelona Hospital Campus, Autonomous University of Barcelona (UAB), Barcelona, Catalonia Spain; 62 Infection in Immunocompromised Pediatric Patients Research Group, Vall d’Hebron Research Institute (VHIR), Vall d’Hebron University Hospital (HUVH), Vall d’Hebron Barcelona Hospital Campus, Barcelona, Catalonia Spain; 63 Department of Biomedicine and Prevention, Tor Vergata University of Rome, Rome, Italy; 64 IRCCS Neuromed, Pozzilli, Italy; 65 Laboratory of Medical Genetics, Translational Cytogenomics Research Unit, Bambino Gesù Children Hospital, IRCCS, Rome, Italy; 66 Department of Molecular Biology and Genetics, Bilkent University, Ankara, Turkey; 67 Laboratory of Immunogenetics of Human Diseases, IdiPAZ Institute for Health Research, University Hospital “La Paz”, Madrid, Spain; 68 Neurometabolic Diseases Laboratory, Bellvitge Biomedical Research Institute (IDIBELL), Barcelona, Catalonia Spain; 69 Center for Biomedical Research On Rare Diseases (CIBERER), ISCIII, Madrid, Spain; 70 Faculdades Pequeno Príncipe, Instituto de Pesquisa Pelé Pequeno Príncipe, Curitiba, Brazil; 71 University of La Sabana, Chía, Colombia; 72 Department of Immunology, University Hospital of Gran Canaria Dr. Negrin, Canarian Health System, Las Palmas de Gran Canaria, Spain; 73 Department of Clinical Sciences, University of Fernando Pessoa Canarias, Las Palmas de Gran Canaria, Spain; 74 Division of Immunology, Transplantation and Infectious Diseases, IRCCS San Raffaele Scientific Institute, Milan, Italy; 75 Specialized Immunology Laboratory of Dr Shahrooei, Sina Medical Complex, Ahvaz, Iran; 76 Department of Microbiology and Immunology, Clinical and Diagnostic Immunology, KU Leuven, Leuven, Belgium; 77 Department of Pediatrics, Mansoura University Children’s Hospital, Mansoura University Faculty of Medicine, Mansoura, Egypt; 78 Hypoxia and Lung, INSERM U1272, Avicenne Hospital, Assistance Publique-Hôpitaux de Paris, Bobigny, France; 79 Department of Life Sciences, School of Science, University of Management and Technology, Lahore, Pakistan; 80 Division of Immunology, Transplantation and Infectious Diseases, IRCCS Ospedale San Raffaele, Milan, Italy; 81 Department of Internal Medicine, Infanta Leonor University Hospital, Madrid, Spain; 82 Department of Neurology, Amsterdam UMC, Amsterdam Neuroscience, Amsterdam, Netherlands; 83 Biosciences Institute, University of São Paulo, São Paulo, Brazil; 84 MNM Bioscience Inc, Cambridge, MA USA; 85 Faculty of Physics, Adam Mickiewicz University, Poznan, Poland; 86 Department of Pediatrics, Immunology Research Laboratory, College of Medicine and King Saud University Medical City, King Saud University, Riyadh, Saudi Arabia; 87 The Genetics Institute, Tel Aviv Sourasky Medical Center, Tel Aviv, Israel; 88 Sackler Faculty of Medicine, Tel Aviv University, Tel Aviv, Israel; 89 Departments of Pediatrics and Microbiology, Immunology, and Molecular Genetics, Division of Immunology, Allergy, and Rheumatology, University of California Los Angeles, Los Angeles, CA USA; 90 Ludwig Institute for Cancer Research, Brussels, Belgium; 91 SIGN Unit, de Duve Institute, Université Catholique de Louvain, Brussels, Belgium; 92 WELBIO (Walloon Excellence in Life Sciences and Biotechnology), Brussels, Belgium; 93 Nuffield Department of Medicine, Ludwig Institute for Cancer Research, Oxford University, Oxford, UK; 94 Department of Pediatrics, Division of Rheumatology/Immunology, Washington University in St. Louis, St. Louis, MO USA; 95 The American Genome Center, Uniformed Services University of the Health Sciences, Bethesda, USA; 96 Department of Anatomy, Physiology & Genetics, Uniformed Services University of the Health Sciences, Bethesda, MD USA; 97 School of Life Sciences, École Polytechnique Fédérale de Lausanne, Lausanne, Switzerland; 98 Swiss Institute of Bioinformatics, Lausanne, Switzerland; 99 Precision Medicine Unit, Lausanne University Hospital and University of Lausanne, Lausanne, Switzerland; 100 Institute for Systems Biology, Seattle, WA USA; 101 Department of Paediatrics and Adolescent Medicine, Queen Mary Hospital, Li Ka Shing Faculty of Medicine, University of Hong Kong, Hong Kong, China; 102 Laboratory of Genetics and Genomics, The Rockefeller University, New York, NY USA; 103 Department of Genetics, Yale University School of Medicine, New Haven, CT USA; 104 Yale Center for Genome Analysis, Yale School of Medicine, New Haven, CT USA; 105 Zukerman Mind Brain Behavior Institute, Columbia University, New York, NY USA; 106 New York Genome Center, New York, NY USA; 107 Department of Biomedicine, Aarhus University, Aarhus, Denmark; 108 Department of Infectious Diseases, Aarhus University Hospital, Aarhus, Denmark; 109 Department of Paediatric Respiratory Medicine, Immunology, and Critical Care Medicine, Charité Universitätsmedizin Berlin, Berlin, Germany; 110 Laboratoire de Biologie Médicale Multisites Seqoia, MG2025, MG2025 Paris, France; 111 Université Paris-Saclay, CEA, Centre National de Recherche en Génomique Humaine (CNRGH), Evry, France; 112 Invitae, San Francisco, CA USA; 113 Unité de Recherche Clinique, Hôpital Bichat, Assistance Publique-Hôpitaux de Paris, Paris, France; 114 Centre d’Investigation Clinique, Hôpital Bichat, Assistance Publique-Hôpitaux de Paris, Paris, France; 115 Département d’immunologie, INSERM Centre d’Immunologie Et Des Maladies Infectieuses CIMI-Paris, Hôpital Pitié-Salpêtrière, Assistance Publique-Hôpitaux de Paris, Sorbonne Université, Paris, France; 116 Département de Santé Publique, Unité de Recherche Clinique PSL-CFX, CIC-1901, Institut Pierre Louis d’Epidémiologie Et de Santé Publique, INSERM, Hôpital Pitié-Salpêtrière, Assistance Publique-Hôpitaux de Paris, Sorbonne Université, Paris, France; 117 Emergency Department, Hôpital Pitié-Salpêtrière, APHP-Sorbonne Université, Paris, France; 118 GRC-14 BIOFAST Sorbonn Université, UMR INSERM 1166, IHU ICAN, Sorbonne Université, Paris, France; 119 Department of Microbiology, Immunology and Transplantation, Laboratory for Inborn Errors of Immunity, KU Leuven, Leuven, Belgium; 120 Laboratory of Host Defenses, NIAID, National Institutes of Health, Bethesda, MA USA; 121 Howard Hughes Medical Institute, New York, NY USA

**Correction: Genome Medicine 15, 22 (2023)**

**https://doi.org/10.1186/s13073-023-01173-8**

The original publication of this article [1] contained an incorrect author name. The incorrect and correct information is listed in this correction article. The original article has been updated.

Incorrect

Alessio Fiorentino

Correct

Alessia Fiorentino
