# Supplementary material for: Rare predicted loss-of-function variants of type I IFN immunity genes are associated with life-threatening COVID-19
Source: Genome Med. 2023 Apr 5;15:22. doi: 10.1186/s13073-023-01173-8 (PMC10074346; doi:10.1186/s13073-023-01173-8)
Supplement: Supplementary file 1 — Additional file 1: Supplementary Methods. Fig S1. Luciferase assay on HEK293T cells transfected with the pGL4.32 luciferase reporter construct and an expression vector for Renilla luciferase together with no vector (mock), EV, WT, or 4 TLR7 variants found in our cohort. After 24 h, transfected cells were left untreated or were treated by incubation with 1 μg/mL R848 for 24 h. These data were established from two independent experiments. The y-axis represents NF-κB transcriptional activity as a percentage of the WT. The x-axis indicates the alleles used for transfection. Fig S2. Age distribution as boxplot and violin plot of the critical COVID-19 cases according to the carrier status of pLOF/bLOF at 15 type I IFN-related loci. Mean age of the patients for each category is shown in red. T-test was used to compare the means, showing a significant difference between non-carriers and carriers of heterozygous or homozygous/hemizygous variants (P = 2.2 × 10^−6^) and between heterozygous carriers and homozygous/hemizygous carriers (P = 0.008). Fig S3. Empirical power of our sample to detect an association at the 2.5 × 10^−6^ exome-wide significance threshold for various relative risks and proportion of carriers of at least one disease causing variant in the general population (PD), as estimated by simulation study (N = 1000 replicates). [file 13073_2023_1173_MOESM1_ESM.docx]

**Rare predicted loss-of-function variants of type I IFN immunity genes**

**are associated with life-threatening COVID-19**

**Supplemental Methods:**

***Sequencing***

The whole-exome (N= 2003 cases and 866 controls) or whole-genome (N=1266 cases and 507 controls) was sequenced at several sequencing centers, including the Genomics Core Facility of the Imagine Institute (Paris, France), the Yale Center for Genome Analysis (USA), Macrogen (USA), Psomagen (USA), the New-York Genome Center (NY, USA), TAGC (USUHS, Bethesda, USA), MNM Bioscience (Poland), Invitae (San Francisco, USA), the Genomic Sequencing Platform Seqoia (France), the Centre National de Recherche en Génomique Humaine (CNRGH, Evry, France), the Genomics Division-ITER of the Canarian Health System sequencing hub (Canary Islands, Spain), the AlJalila Genomics Center (Dubai). Libraries for WES were generated with the Twist and Twist Plus Human Core Exome Kit, the xGen Exome Research Panel from Integrated DNA Technologies (IDT; xGen V1 and V2), Agilent SureSelect (Clinical Research exome V2, Human All Exon V6 and V7) panels, the SeqCap EZ MedExome Kit from Roche, the Nextera Flex for Enrichment-Exome kit, the Illumina TruSeq Exome panel and WES custom target enrichlent probes. Massively parallel sequencing was performed on HiSeq 4000, HiSeq 2500, NextSeq 550 or NovaSeq 6000 systems (Illumina). For 3363 samples (2493 critical cases and 870 controls,), the FASTQ files were centralized and processed in the laboratory of Human Genetics of Infectious Diseases (HGID). Raw reads were aligned and mapped to the human reference genome assembly hg19 – NCBI build 37 using the Burrows-Wheeler Aligner (BWA). Post alignment processing procedures from Genome Analysis Software Kit (GATK version 3.4-46) best-practice pipeline were applied to minimize eventual artifacts that may affect the quality of WES/WGS data. PCR duplicates were removed with Picard tools (broadinstitute.github.io/picard/). The GATK base quality score recalibrator (BQSR) was applied to correct sequencing artifacts. Individual genomic variant call files (gVCF) were generated with GATK HaplotypeCaller, and joint genotyping was performed with GATK GenotypeGVCFs in the interval intersecting all the main WES capture kits of ±50 bp. We also searched for rare homozygous or hemizygous deletions from the NGS data using HMZDelFinder-opt [1]. For the remaining 1280 samples (777 cases and 503 controls), raw data were processed separately by each sequencing hub using either GATK best-practice pipeline or Illumina DRAGEN pipeline. For those samples, only single sample VCF files were centralized in the laboratory of Human Genetics of Infectious Diseases. VCF files were merged together and with the multi-sample VCF generated by the HGID pipeline using BCFtools v1.9 (<http://github.com/samtools/bcftools>), assuming that genotypes at missing sites were homozygous reference. Only variants located in the interval intersecting all the main WES capture kits ±50 bp were kept.

***Quality Control***

Per-sample quality control (QC) metrics (mean variant depth, number of indels, transitions/transversions rate, number of heterozygous, reference/non reference homozygous ratio) were generated using BCFtools v1.9 (<http://github.com/samtools/bcftools>) and samples outlier on the QC metric distributions were excluded. We estimated the kinship coefficient between all pairs of samples using the related option of the king software and excluded first degree relatives. Sex was inferred from the coverage of the X and Y chromosomes and samples with inconsistencies between the reported and inferred sex were excluded. Sample genotypes with a coverage of <8×, a genotype quality of <20, or a ratio of reads for the less covered allele (reference or variant allele) over the total number of reads covering the position (minor read ratio) of <20% for heterozygous calls were filtered out. We excluded variant sites (i) with a call rate of <50% in gnomAD genomes and exomes, (ii) with a non-PASS filter in the gnomAD database, (iii) falling in low-complexity or decoy regions, (iv) that were multiallelic with more than four alleles,(v) spanning more than 20 nucleotides, (vi) with more than 5% missing genotypes in our cohort with raw data available, (vii) with differential missingness between cases and controls (P-value <10^-4^), (viii) blacklisted using our blacklist procedure [2]. We generated four blacklists (i.e. variants absent from Gnomad v2.1 while present at frequency > 1% in our dataset) according to the type of data available (FASTQ vs VCF only) and the type of sequencing (WES vs WGS). Variants falling in at least one of the four blacklists were excluded. For variants identified in the 13 type I IFN-related influenza susceptibility loci, manual review of aligned reads for variant confirmation was done with The Integrative Genomics Viewer [3].

***Variant annotation***

Variant effects were predicted with the Ensembl Variant Effect Predictor and the Ensembl GRCh37.75 reference database, retaining the most deleterious annotation per gene obtained from Ensembl protein-coding transcripts overlapping with RefSeq transcripts. The Combined Annotation Dependent Depletion (CADD) score [4] was used to predict the potential deleteriousness of the variants. However, the CADD score for known pathogenic mutations is highly variable from one gene to another and it may be useful to consider specific gene thresholds rather than a fixed threshold; to do so, we used the MSC (“Mutation Significance Cut-off”) which is defined for a given gene as the lower limit of the confidence interval (95%) of the CADD score of all its known pathogenic mutations [5]. Thus, variants with a CADD score higher than the MSC are more likely to lead to an altered phenotype than variants with a CADD lower than the MSC. In addition, allele frequencies in exomes and genomes were retrieved for each variant from the Genome Aggregation Database (GnomAD v2.1).

***Simulation study***

We performed a simulation study to assess the empirical p-value for the test of enrichment in rare pLOF variants in cases at 13 selected loci. Each of the 13 genes were matched to a set of genes with similar pLI [6] and CoNeS [7] score in order to account for the level of gene tolerance to pLOF and of overall negative selection, respectively (Supplemental Table 12). The pLI was developed with ExAC [6] and estimates the probability that a gene falls into the class of LoF-haploinsufficient genes. However, the ability to comprehensively characterize the degree of selection against pLoF variants is limited, as for small genes, the expected number of mutations is very low [6,8]. We recently developed an integrative score, denoted as consensus negative selection (CoNeS) [7], to approximate the strength of negative selection. CoNeS was obtained through a standardized (i.e., mean of 0 and SD of 1) projection on the first principal component of seven measures including pLI, and accounting for both interspecies and intraspecies information. Low CoNeS values are associated with stronger selection constraints, and correlates with higher pLI values. We considered 9 bins of pLI (including a bin for genes with non available values) and 9 bins of CoNeS (including a bin for genes with non available values). The number of genes in each category is provided in Supplemental Table 12, as well as the category to which belongs each of the 13 genes. In each of the 100,000 replicates, we randomly selected 13 loci with similar pLI and CoNeS values. We tested for an enrichment of pLOF variants with frequency < 10^-3^ at those 13 randomly selected loci in our 3269 critical cases as compared with our 1373 infected controls. The analyses were performed by means of LRT with Firth’s bias correction under a codominant model of inheritance, and were adjusted on sex, age (in years) and five first PCs of the PCA. To estimate the empirical p-value, we extracted the number of replicates with enrichment of pLOF variants in cases that gave a lower p-value than the one observed for the enrichment in rare pLOF variants at 13 type I IFN-related influenza susceptibility loci (P = 2.1x10^-4^), and divided it by the total number of replications (100,000).

**Genome-wide weighted burden analysis of rare and common variants**

We performed a genome-wide gene-based weighted burden association test including both common and rare candidate coding variants. We implemented a weighted burden test in the logistic regression framework, following a strategy similar to that reported in [9]. For each individual, we computed a genetic score as the sum of the number of alleles at each variant weighted according to the variant’s frequency. We used the weight function proposed in [10] and defined as:

W_i_ = (4f-4)q_i_²-(4f-4)q_i_+f,

where f = 20 and q_i_ is the minor allele frequency of variant I observed in the case-control sample. This function gives a weight of 1 to common variants with minor allele frequency of 0.5 and ~20 to rare variants with frequency below 0.001. Missing genotypes were replaced by the expected number of minor alleles derived from the minor allele frequency of the variant in the case-control sample.

**Power simulation study**

We evaluated the power to detect an exome-wide significant association in our sample for various relative risks and frequency of disease allele carriers. To do this, we simulated a large reference population assumed to be infected with SARS-CoV-2 with a proportion PD of carriers of at least one disease causing variant. We calculated the probability of developing a critical COVID-19 pneumonia for non-carriers (f0) and for carriers (f1) of at least one disease causing variant as follows:

$$f0= \frac{\mathrm{ICR}}{1-PD+RRxPD}, and$$

$$f1=f0 x RR$$

Where ICR is the age-specific infection critical rate taken from [11], and RR is the relative risk of critical COVID-19 pneumonia for carriers versus non-carriers. We used bins of 10 years from 0 to more than 80 years ([0-9], [10-19], [20-29], [30-39],[40-49], [50-59], [60-69], [70-79], 80+). PD varied from 0.001 to 0.01 and the RR varied from 2 to 10.

In each age category, we randomly sampled 1,000 times the same number of cases and controls observed in our sample. In each replicate, we tested for the association between the carrier status and the case-control phenotype by mean of Firth’s bias corrected logistic regression using age as covariate. We estimated the power at the 2.5x10^-6^ exome-wide significance cut-off as the proportion of replicate giving a p-value of association < 2.5x10^-6^.


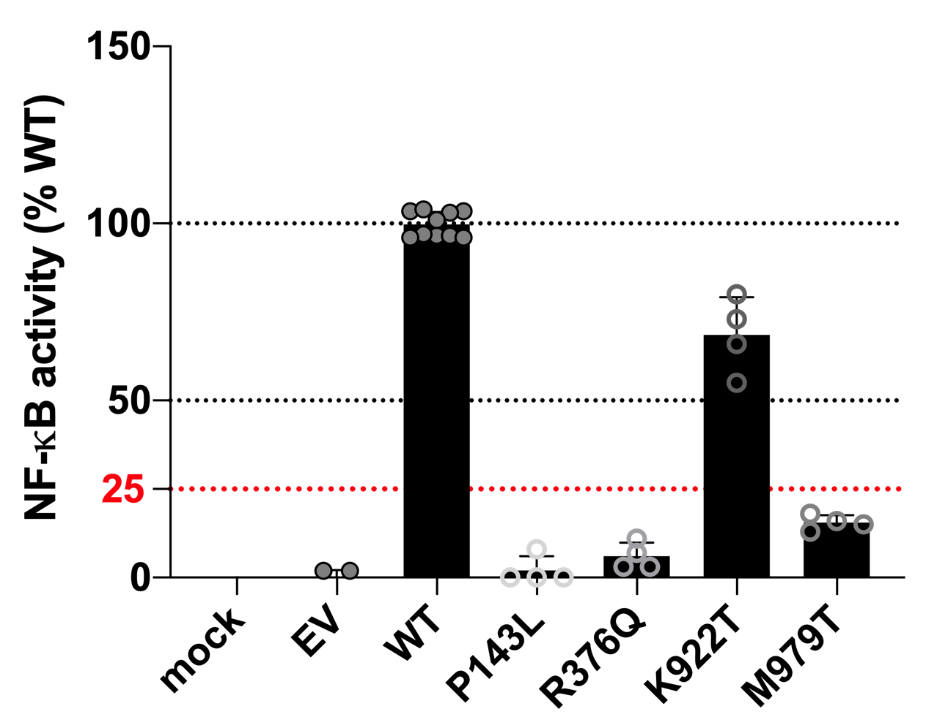


**Fig S1.** Luciferase assay on HEK293T cells transfected with the pGL4.32 luciferase reporter construct and an expression vector for *Renilla* luciferase together with no vector (mock), EV, WT, or 4 *TLR7* variants found in our cohort. After 24 hours, transfected cells were left untreated or were treated by incubation with 1 μg/mL R848 for 24 hours. These data were established from two independent experiments. The *y*-axis represents NF-κB transcriptional activity as a percentage of the WT. The *x*-axis indicates the alleles used for transfection.

**
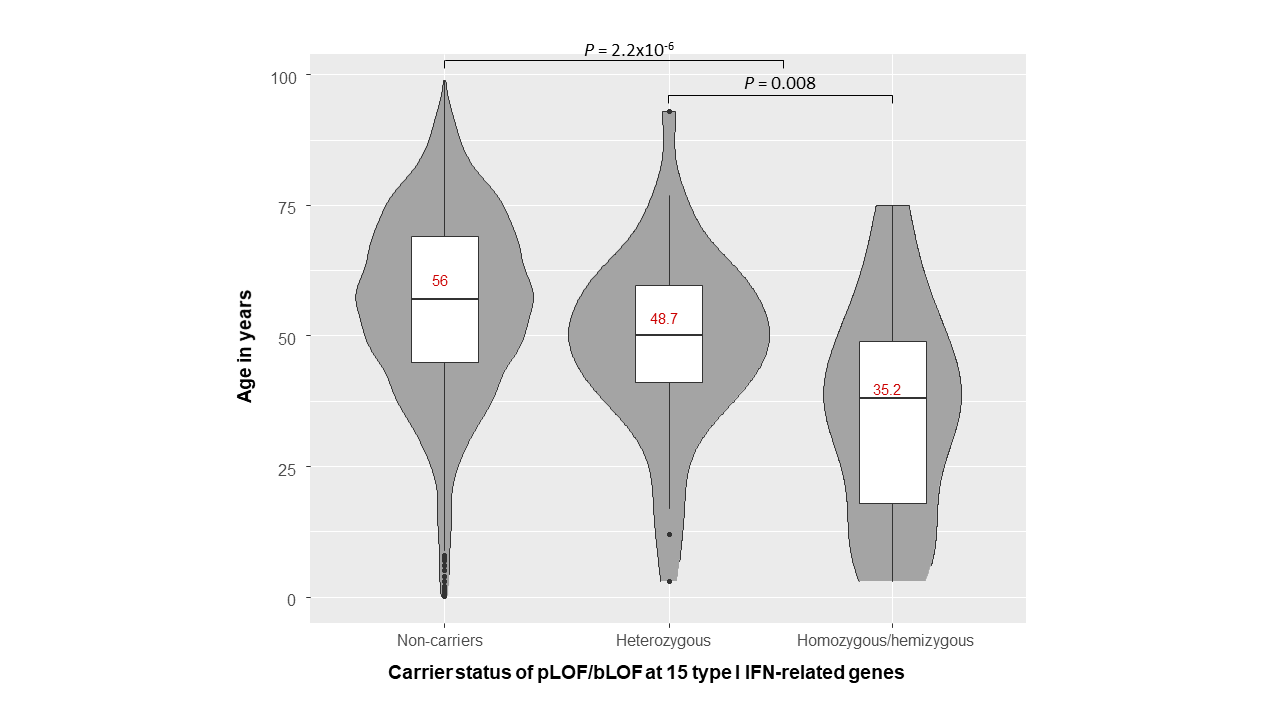
**

**Fig S2.** Age distribution as boxplot and violin plot of the critical COVID-19 cases according to the carrier status of pLOF/bLOF at 15 type I IFN-related loci. Mean age of the patients for each category is shown in red. T-test was used to compare the means, showing a significant difference between non-carriers and carriers of heterozygous or homozygous/hemizygous variants (*P* = 2.2x10^-6^) and between heterozygous carriers and homozygous/hemizygous carriers (*P* = 0.008).


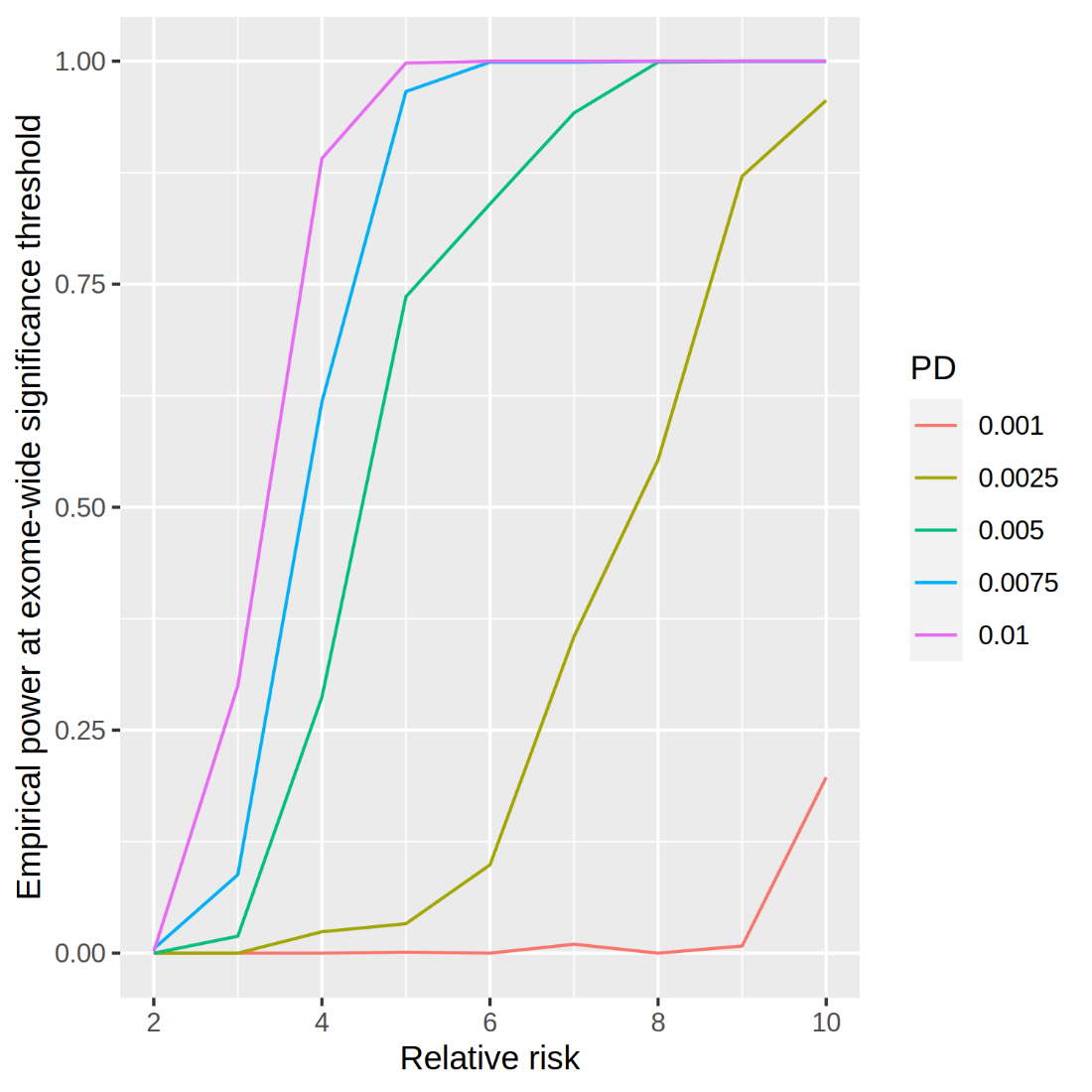


**Fig S3**. Empirical power of our sample to detect an association at the 2.5x10^-6^ exome-wide significance threshold for various relative risks and proportion of carriers of at least one disease causing variant in the general population (PD), as estimated by simulation study (N=1000 replicates).

**References**

1. Bigio B, Seeleuthner Y, Kerner G, Migaud M, Rosain J, Boisson B, et al. Detection of homozygous and hemizygous complete or partial exon deletions by whole-exome sequencing. NAR Genom Bioinform. 2021;3:lqab037.

2. Maffucci P, Bigio B, Rapaport F, Cobat A, Borghesi A, Lopez M, et al. Blacklisting variants common in private cohorts but not in public databases optimizes human exome analysis. Proc Natl Acad Sci U A. 2019;116:950–9.

3. Robinson JT, Thorvaldsdottir H, Winckler W, Guttman M, Lander ES, Getz G, et al. Integrative genomics viewer. Nat Biotechnol. 2011;29:24–6.

4. Rentzsch P, Witten D, Cooper GM, Shendure J, Kircher M. CADD: predicting the deleteriousness of variants throughout the human genome. Nucleic Acids Res. 2019;47:D886–94.

5. Itan Y, Shang L, Boisson B, Ciancanelli MJ, Markle JG, Martinez-Barricarte R, et al. The mutation significance cutoff: gene-level thresholds for variant predictions. Nat Methods. 2016;13:109–10.

6. Lek M, Karczewski KJ, Minikel EV, Samocha KE, Banks E, Exome Aggregation Consortium, et al. Analysis of protein-coding genetic variation in 60,706 humans. Nature. 2016;536:285–91.

7. Rapaport F, Boisson B, Gregor A, Béziat V, Boisson-Dupuis S, Bustamante J, et al. Negative selection on human genes underlying inborn errors depends on disease outcome and both the mode and mechanism of inheritance. Proc Natl Acad Sci. 2021;118:e2001248118.

8. Karczewski KJ, Francioli LC, Tiao G, Cummings BB, Alföldi J, Wang Q, et al. The mutational constraint spectrum quantified from variation in 141,456 humans. Nature. 2020;581:434–43.

9. Curtis D. A weighted burden test using logistic regression for integrated analysis of sequence variants, copy number variants and polygenic risk score. Eur J Hum Genet. 2019;27:114–24.

10. Curtis D. A rapid method for combined analysis of common and rare variants at the level of a region, gene, or pathway. Adv Appl Bioinforma Chem. 2012;1.

11. Herrera-Esposito D, de los Campos G. Age-specific rate of severe and critical SARS-CoV-2 infections estimated with multi-country seroprevalence studies. BMC Infect Dis. 2022;22:311.
